# Transcriptome Profiling and Genome-Wide Association Studies Reveal *GSTs* and Other Defense Genes Involved in Multiple Signaling Pathways Induced by Herbicide Safener in Grain Sorghum

**DOI:** 10.3389/fpls.2019.00192

**Published:** 2019-03-08

**Authors:** You Soon Baek, Loren V. Goodrich, Patrick J. Brown, Brandon T. James, Stephen P. Moose, Kris N. Lambert, Dean E. Riechers

**Affiliations:** ^1^Department of Crop Sciences, University of Illinois at Urbana-Champaign, Urbana, IL, United States; ^2^Jerseyville Research Center, Monsanto Company, Jerseyville, IL, United States; ^3^Department of Plant Sciences, University of California, Davis, Davis, CA, United States; ^4^HudsonAlpha Institute for Biotechnology, Huntsville, AL, United States

**Keywords:** herbicide safeners, transcriptome analysis-RNAseq, glutathione *S*-transferases, plant defense, defense signaling network, dhurrin metabolism, detoxification

## Abstract

Herbicide safeners protect cereal crops from herbicide injury by inducing genes and proteins involved in detoxification reactions, such as glutathione *S*-transferases (GSTs) and cytochrome P450s (P450s). Only a few studies have characterized gene or protein expression profiles for investigating plant responses to safener treatment in cereal crops, and most transcriptome analyses in response to safener treatments have been conducted in dicot model species that are not protected by safener from herbicide injury. In this study, three different approaches were utilized in grain sorghum (*Sorghum bicolor* (L.) Moench) to investigate mechanisms involved in safener-regulated signaling pathways. An initial transcriptome analysis was performed to examine global gene expression in etiolated shoot tissues of hybrid grain sorghum following treatment with the sorghum safener, fluxofenim. Most upregulated transcripts encoded detoxification enzymes, including P450s, GSTs, and UDP-dependent glucosyltransferases (UGTs). Interestingly, several of these upregulated transcripts are similar to genes involved with the biosynthesis and recycling/catabolism of dhurrin, an important chemical defense compound, in these seedling tissues. Secondly, 761 diverse sorghum inbred lines were evaluated in a genome-wide association study (GWAS) to determine key molecular-genetic factors governing safener-mediated signaling mechanisms and/or herbicide detoxification. GWAS revealed a significant single nucleotide polymorphism (SNP) associated with safener-induced response on chromosome 9, located within a phi-class *SbGST* gene and about 15-kb from a different phi-class *SbGST*. Lastly, the expression of these two candidate *SbGSTs* was quantified in etiolated shoot tissues of sorghum inbred BTx623 in response to fluxofenim treatment. *SbGSTF1* and *SbGSTF2* transcripts increased within 12-hr after fluxofenim treatment but the level of safener-induced expression differed between the two genes. In addition to identifying specific GSTs potentially involved in the safener-mediated detoxification pathway, this research elucidates a new direction for studying both constitutive and inducible mechanisms for chemical defense in cereal crop seedlings.

## Introduction

Plants have the unique capability of stimulating selective signaling pathways in response to diverse stimuli, including natural and synthetic compounds as well as abiotic and biotic stresses. Subsequently, plants activate specific defense, and detoxification mechanisms for survival and/or adaptation (Cole and Edwards, 2000; Goda et al., 2004; Riechers et al., 2010; Züst and Agrawal, 2017). Plant defense mechanisms elicited under biotic and abiotic stress are typically activated through plant hormone-mediated signaling (Bari and Jones, 2009), which may include the upregulation of detoxification enzymes that recognize and metabolize a diverse range of natural and synthetic compounds (*xenobiotics*; Edwards et al., 2000; Cummins et al., 2011). In addition to direct metabolism of xenobiotic substrates, other important detoxification mechanisms also include transport and compartmentalization of non-phytotoxic, polar metabolites (Coleman et al., 1997; Riechers et al., 2010; Ramel et al., 2012).

One of the most well-studied xenobiotic responses is the herbicide detoxification pathway, consisting of a complex, multistep process that metabolizes different herbicide substrates. These detoxification processes occur in three sequential phases: Phase I involves hydrolysis or oxidation and Phase II involves conjugation with endogenous sugars or reduced glutathione (GSH). During Phase III, the conjugates are exported from the cytosol and sequestrated within the vacuole. Interestingly, herbicide detoxification reactions can also be induced by certain synthetic compounds called herbicide safeners. Safeners are non-phytotoxic chemical compounds that selectively increase tolerance to herbicides in certain monocotyledonous crop species, such as maize, wheat, rice, and grain sorghum, without reducing herbicide susceptibility in target weeds (Hatzios and Hoagland, 1989; Riechers et al., 2010). The predominant mechanisms for safener-induced plant detoxification include an enhanced rate of herbicide metabolic detoxification and/or sequestration (Davies and Caseley, 1999; Hatzios and Burgos, 2004; Riechers et al., 2010; Edwards et al., 2011).

Key proteins/enzymes in these detoxification pathways include esterases, amidases, oxidases, and cytochrome P450 monooxygenases (P450s) in Phase I, glutathione *S*-transferases (GSTs) and UDP-dependent glucosyltransferases (UGTs) in Phase II, and ATP-binding cassette (ABC)-transporters in Phase III (Kreuz et al., 1996). Several studies have shown that safeners induce the expression of genes encoding these key enzymes (reviewed by Riechers et al., 2010). Grain sorghum (*Sorghum bicolor*) has previously demonstrated a massive induction of herbicide-metabolizing GSTs in response to several herbicide safeners (Fuerst and Gronwald, 1986; Gronwald et al., 1987; Gronwald and Plaisance, 1998). This biochemical reaction of GST-mediated detoxification through conjugation with GSH is well characterized (Dixon et al., 2002), but the precise molecular mechanism for the induction of GSTs and other defense enzymes is poorly understood (Riechers et al., 2010). Previously, proteomic approaches identified proteins involved in safener responses in the model grass *Aegilops tauschii*, a diploid wheat species (Zhang and Riechers, 2004; Zhang et al., 2007). These studies detected large increases of GST expression in coleoptile tissues and identified several new safener-inducible proteins, including 12-oxophytodienoate reductases (OPRs). The coleoptile is a transient organ and the outermost structure of emerging grass shoots exposed to environmental factors and stressors, analogous to the leaf epidermis (Javelle et al., 2011). The coleoptile protects inner leaf tissues through both structural and chemical defense mechanisms. Taken together, these results suggest that safeners may coordinately induce the expression of detoxification enzymes in a tissue-specific manner in cereal crop seedlings (Riechers et al., 2010; Riechers and Green, 2017).

Several hypotheses for potential signaling mechanism(s) underlying safener-regulated detoxification reactions have been proposed, based mainly on research performed with model dicots (Rishi et al., 2004; Behringer et al., 2011; Skipsey et al., 2011). Expression of several safener-induced genes related to detoxification were dependent on TGA transcription factors and/or NON-Expressor of PR-1 (NPR1) (Behringer et al., 2011), the key regulators of salicylic acid (SA) synthesis (Zhang et al., 1999; Uquillas et al., 2004; Mueller et al., 2008). Additionally, linkages have been hypothesized between safener-mediated detoxification and signaling pathways utilizing oxylipins, which are oxidation products of membrane-derived polyunsaturated fatty acids (Mueller et al., 2008; Mosblech et al., 2009). A dramatic decrease in safener-induced *GST* expression levels was measured in root cultures of *Arabidopsis fad* mutants unable to synthesize oxylipins, implying a direct link between safener-regulated responses and the oxylipin signaling pathway (Skipsey et al., 2011). Recent research to functionally test this hypothesis showed that oxylipin treatment induced *GST* expression in rice plants but did not confer a safening phenotype from herbicide injury (Brazier-Hicks et al., 2018), suggesting that the pathway(s) mediating safener responses contain signaling components in addition to oxylipins.

Safeners hold great promise for discovering and commercializing novel agrochemicals for crop protection (Riechers and Green, 2017) as well as understanding the regulation of plant defense mechanisms and signaling pathways. However, detailed molecular mechanisms are limited in the literature pertaining to regulation of gene expression by safeners in cereal crops where a clear phenotypic response is observed (Riechers et al., 2010). To test the hypothesis that safeners induce expression of numerous genes involved in detoxification and signaling pathways, which may involve oxylipins and/or phytohormones, our objectives were to: (1) investigate global transcriptome expression of the safener response in etiolated grain sorghum shoots by RNAseq, (2) identify genes responsible for natural or safener-induced herbicide tolerance via a genome-wide association study (GWAS), and (3) investigate the expression of candidate safener upregulated genes (identified by GWAS) at different time points, using validated reference genes in etiolated shoot tissues. This study provides novel insights pertaining to how safeners reprogram the plant transcriptome and elucidates mechanisms involved in detoxification reactions in grain sorghum seedlings.

## Materials and Methods

### Plant Materials, Growth Conditions, and Treatments

Sorghum seeds (commercial sorghum hybrid 7431; Advanta Seeds, USA) were surface sterilized with 5% (v/v) sodium hypochlorite for 5 min and rinsed with deionized water 3 times for 5 min each. Twenty-five sterilized seeds were planted at the depth of 3.8 cm in each 4 × 4 cm pot containing vermiculite, and three pots were prepared for each of the following treatments. Each pot was watered with 150 mL deionized water applied via soil drench, covered with aluminum foil, then incubated in a growth chamber at 27°C in the dark for 42 h. A non-treated control (0.4% DMSO only) or safener (20 μM fluxofenim) treatment was then applied to each pot in 50 mL deionized water. After 12 h, etiolated shoot tissues were harvested (1–2 cm above the seed) and frozen in liquid nitrogen, then stored at −80 ° C until RNA extraction. Whole-plant responses to fluxofenim, *S*-metolachlor (herbicide), and the combination treatment 14 d after application are shown in Supplementary Figure 1.

### RNA Extraction, Library Construction, and Transcriptomic Analysis

To test the whole sorghum transcriptome response to safener, frozen shoot samples were ground in liquid nitrogen and total RNA was isolated from 500 mg of shoot material using previously described methods (Xu et al., 2002) with Trizol reagent (Invitrogen). The concentration and purity of extracted RNA were determined with NanoDrop 1,000 spectrophotometer (Thermo Scientific, Waltham, MA, USA) and a Qubit 2.0 fluorometer (Invitrogen, USA). Samples with concentrations ranging between 50 and 100 ng/μL and an A_260_/A_280_ >1.95 were utilized for library preparation. To visually evaluate rRNA integrity, total RNA (1 μg) was denatured at 55 ° C in the presence of formamide and formaldehyde and visualized on 1% EtBr-stained agarose gels containing 0.4 M formaldehyde as previously described (Riechers et al., 2003).

Three individual pots for each of the two treatments were treated as biological replicates, resulting in a total of six libraries. RNA samples were obtained and libraries were constructed from two independent experiments, in which the first experiment consisted of two libraries (one replicate per treatment) and the second experiment consisted of four libraries (two replicates per treatment) for a total of three biological replicates per treatment (i.e., with or without safener). RNA quality control and library preparation analyses were performed by the High-Throughput Sequencing and genotyping unit at the University of Illinois Biotechnology center (http://www.biotech.illinois.edu). Briefly, stranded, single-read libraries of total RNA were generated for each sample using the Illumina Truseq Stranded RNAseq sample preparation kits. The libraries were quantitated by qPCR and sequenced on one lane for 101 cycles from one end of the fragments on a HiSeq2000 for the first experiment and Hiseq2500 for the second experiment, using a TrueSeq SBS sequencing kit (version 3 or 4, respectively). Fastq files were generated and demultiplexed with the bcl2fasq v2.17.1.14 conversion software (and the Illumina software Casava 1.8.2) for the first experiment). Two RNAseq libraries from the first experiment were sequenced on a single lane that produced 100-bp single-end reads, and four RNAseq libraries from the second experiment were sequenced separately on a single lane.

The published sorghum reference genome (*S. bicolor v3.1.1*) and genome annotations for analyzing differentially expressed transcripts were downloaded from Phytozome (phytozome.jgi.doe.gov). Prior to mapping and assembly, total reads were filtered to remove reads with adapters, low-quality reads (mean Q < 10), incomplete reads (< 50-bp), or repetitive reads with mutual information score >0.5 using Trimmomatic/0.36-Java-1.8.0_121. Both reads in a pair were removed if either one of them failed the filters. On average, <1% of all reads failed to pass the filter. Clean reads were then mapped to the reference genome using the STAR/2.5.3a-aligner with default parameters. On average across libraries, 89% of reads mapped uniquely to the reference genome. Read numbers that mapped to each gene were counted by featureCounts v.1.4.5-pl under the subread/1.5.0 package. The Illumina RNAseq datasets analyzed for this study have been deposited in SRA database with the accession number of PRJNA490688 (https://www.ncbi.nlm.nih.gov/bioproject/PRJNA490688).

All tests of significance for differential expression were analyzed with a linear model implemented by the limma package (Smyth, 2005; Law et al., 2014) and modules from the edgeR package (Robinson et al., 2010) in R (R Development Core Team, 2011), in which the TMM normalization method was used to adjust expression values to a common scale. Gene expression levels were calculated using counts-per-million (CPM) and transformed to log_2_-counts per million (log-CPM). Global changes in differentially expressed genes (DEGs) were identified if transcripts showed differences in expression >2 log (log_2_)-fold, with false-discovery rate (FDR)-corrected *p*-values (q-values) <0.05. The genome-wide patterns of expression were visualized through a principal component analysis (PCA) of the normalized mean read counts per gene (CPM in the library) to estimate the possible variances among libraries, since libraries from two independent experiments were combined. Major biological and molecular functions of DEGs were determined by Gene Othology (GO) enrichment analysis. GO was determined using annotations from agriGO (Du et al., 2010) and as described in Fracasso et al. (2016). Each unique gene within a GO annotation was allowed to contribute to the enrichment of that category. Enrichment analysis of Kyoto Encyclopedia of Genes and Genomes (KEGG; http://www.genome.ad.jp/kegg/; Kanehisa and Goto, 2000) were performed using KEGG Orthology IDs provided by Phytozome.

### Genome Wide Association Study, Phenotyping, and Genotyping Sorghum Inbred Lines

Sorghum inbred lines were from the Sorghum Conversion Program (Thurber et al., 2013). Three independent trials replicated in time (i.e., biological replicates) were conducted using a different randomization scheme in each trial. Flats for trials one and two were planted in a soil, peat, sand mix at a ratio of 1:1:1, whereas flats in trial three were planted in Metro Mix 900 (BFG Supply Co., USA) series soil. Flats contained 24 cells of randomized sorghum genotypes in unsafened/safened pairs plus inbred BTx623 as a control-check in each flat. Safened seeds were treated with fluxofenim (Sigma-Aldrich, St. Louis, MO, USA) at the rate of 0.4 g kg^−1^ seed. Unsafened seeds were not subjected to treatment. Sorghum seeds were planted 3.5 cm deep and 1 cm apart in the flats, bottom watered, and allowed to sit under greenhouse conditions for 24 h before the herbicide treatment was applied. Flats were planted in pairs; one to receive a herbicide treatment and the other to serve as the control (sprayed with water only). For trials one and two, the herbicide *S*-metolachlor was applied at a rate of 2.5 kg ha^−1^ to the soil of the herbicide flats. Herbicide treatments were applied using a Generation III Research Sprayer (DeVries Manufacturing, USA) with a moving-nozzle, compressed air research spray chamber with an adjustable platform and equipped with a TeeJet 80015EVS even flat-spray nozzle. The nozzle was maintained at ~35 cm above the flat and the sprayer was calibrated to deliver 185 L ha^−1^ at 275 kPa. For trial three, *S*-metolachlor was applied at a concentration of 37 μM in a 40 mL solution as a soil drench treatment to each individual flat cell using a 50 mL syringe to ensure that an optimal herbicide response was achieved. Greenhouse conditions for trials one and three were set at 28/22°C day/night with a 16/8-hr photoperiod. Greenhouse conditions for trial two were set at 24/22°C day/night with a 16/8-hr photoperiod. Sorghum seedlings were overhead watered daily. After two weeks, seedlings were harvested at the soil level, then total seedling fresh weights and numbers of emerged seedlings were recorded to quantitatively evaluate herbicide injury. Germination rate in the herbicide-untreated tray was monitored for quality control, and data from one week in the first trial were discarded due to low germination. Data for individual genotypes with very poor seed quality, defined as <25% germination in the herbicide-untreated tray, were also discarded.

The four treatment conditions (+/– herbicide; +/– safener) enabled the analysis of four different contrasts. However, only results from the contrast of greatest interest, the “safener-induced response” (safener-treated vs. safener-untreated in the presence of herbicide), are presented. Using data from all three trials, a single predicted value for each sorghum inbred line was estimated via regression using ASREML-R, where the model included trial, week within trial, tray-pair within week within trial, and inbred line as random effects. Additionally, the inbred sorghum line BTx623, the genotype used to create the sorghum reference genome (Paterson et al., 2009), was included as a control in each tray throughout the experiment, and the mean phenotypic value of BTx623 in the planting for each week was tested as a fixed effect covariate to improve model fit as measured by Bayesian Information Criterion (BIC).

Genotyping-by-sequencing was used to generate genome-wide SNP data for the 761 inbred sorghum genotypes evaluated in this study (Thurber et al., 2013). There were 100,610 SNPs overall, but SNPs with a minor allele frequency below 0.026 and identical SNPs within 64-bp of each other were excluded, leaving 60,167 SNPs for GWAS. A GWAS was performed using the genomic association and prediction-integrated tool (GAPIT) in R (Lipka et al., 2012) using a (non-compressed) mixed linear model (MLM) with the population parameters previously determined (P3D) method. SNPs with FDR-corrected *p*-values (q-values) <0.05 were then compared to the sorghum reference genome and paired with closely-associated genes. Unadjusted *p*-values were displayed as a Manhattan plot, and candidate genes identified through GWAS were selected for further quantitative expression analysis by RT-qPCR.

### Primer Specificity, Determining Suitable Reference Genes, and *SbGST* Expression Analysis in Sorghum Inbred BTx623

Semi-quantitative RT-PCR was performed to determine target *SbGST* gene and candidate reference gene primer specificity and amplicon size. Sequences of two *SbGST* candidate genes identified through GWAS were analyzed for gene-specific primer (GSP) design using Primer3 software and BLAST. Primer sequences for eight candidate reference genes, which had previously displayed stable expression in various sorghum tissues and organs (Zhang et al., 2013; Reddy et al., 2016), were redesigned to meet specific criteria for RT-qPCR analysis in etiolated sorghum shoot tissue. The candidate *SbGST* GSPs and reference gene primers were required to meet the following stringent parameters: melting temperature (T_m_) of 60–63°C, primer lengths of 20–25 bp, guanine-cytosine content 45–55%, amplicon length of 100–250 bp, and the absence of stable hairpins and dimers, determined using the OligoAnalyzer 3.1 tool (Integrated DNA Technologies, USA).

Plant growth conditions and RNA extractions were performed as described previously under “Transcriptomic Analysis,” except the safener treatment was 10 μM fluxofenim and only sorghum inbred BTx623 was used. First-strand cDNA synthesis was performed with 500 ng total RNA using the Maxima H-minus cDNA synthesis kit (Thermo Scientific, Waltham, MA, USA) following the manufacturer's protocol. The following amplification program was used with 1 μL first-strand cDNA reaction for semi-quantitative RT-PCR with a PTC-200 Pellier Thermal Cycler (MJ Research Inc., USA): initial denaturation at 95°C for 4.5 min, then 30 amplification cycles of 95°C for 30 s and 62°C for 1 min, followed by a final extension at 72°C for 5 min. RT-PCR products were separated and visualized on 1.8% agarose gels stained with EtBr. To test primer specificity of two candidate *SbGST* genes, synthetic gene plasmids were synthesized using GeneArt Gene Synthesis (Invitrogen, USA). Each plasmid was synthesized using the entire coding region of the corresponding candidate gene. Semi-quantitative RT-PCR was conducted as above using 1 μL of a 10 ng/μL plasmid solution with both sets of primers to test for specificity of amplification at varying annealing temperatures.

For each candidate reference gene, standard curves were determined by qPCR using 10-fold dilution series over five dilution points of pooled cDNA as a template using the linear regression model (Pfaffl et al., 2004). Primer efficacy for the standard curves was calculated in the SDS 2.3 software (Applied Biosystems, USA). Gene expression stability of the seven candidate reference genes (Supplementary Table 1) was estimated using four statistical algorithms: geNorm (Vandesompele et al., 2002), NormFinder (Andersen et al., 2004), BestKeeper (Pfaffl et al., 2004), and the comparative ΔCt method (Silver et al., 2006). RefFinder was used to compare and integrate the ranking of the tested candidate reference genes (Xie et al., 2012). GeNorm was used to determine the gene expression stability value (M) with a cutoff value for M of 1.5 (Vandesompele et al., 2002). The *CYP* gene was omitted from further RT-PCR analysis due to the inability to design GSPs and form a product.

RT-qPCR was conducted to analyze expression of two *SbGSTs* using a 7900 HT Sequence Detection System (Applied Biosystems, USA) and reactions performed in 20 μL volumes following the manufacturer's protocol (Power Syber® Green RNA-to-C_T_™ 1-Step Kit; Applied Biosystems, USA). The following program was used for qRT-PCR: 48°C for 30 min, 95°C for 10 min, then 40 cycles at 95°C for 15 s, 62°C for 1 min, and a melting curve at 95°C for 15 s and 62°C for 15 s. Each sample was analyzed in three technical replicates and mean Cq values were calculated. Reverse-transcription negative controls were included to ensure the absence of genomic DNA in the template. Dissociation curves for each reaction were analyzed to ensure only one replicon was amplified. Safener-induced gene expression for each *SbGST* gene was calculated relative to transcript levels in the unsafened control samples (per genotype and time after treatment) and normalized using three reference genes (*GTPB, SAND*, and *EIF4a*; described below) using the 2^−ΔΔCt^ method. For quantitative analysis of *SbGSTF1* and *SbGSTF2* expression in inbred BTx623, expression data represent the combined results from three independent experiments (i.e., biological replicates), with three technical replicates per sample. ANOVA followed by Tukey's multiple comparison test and LSD (α = 0.05) using PROC GLM in SAS (Release 9.2) was conducted to determine significant differences in gene expression among sorghum genotypes at each harvest time point.

## Results

### Transcriptome Profiling of Responses to Safener Treatment in Commercial Sorghum Hybrid 7431 Shoots

**Figure 1:**
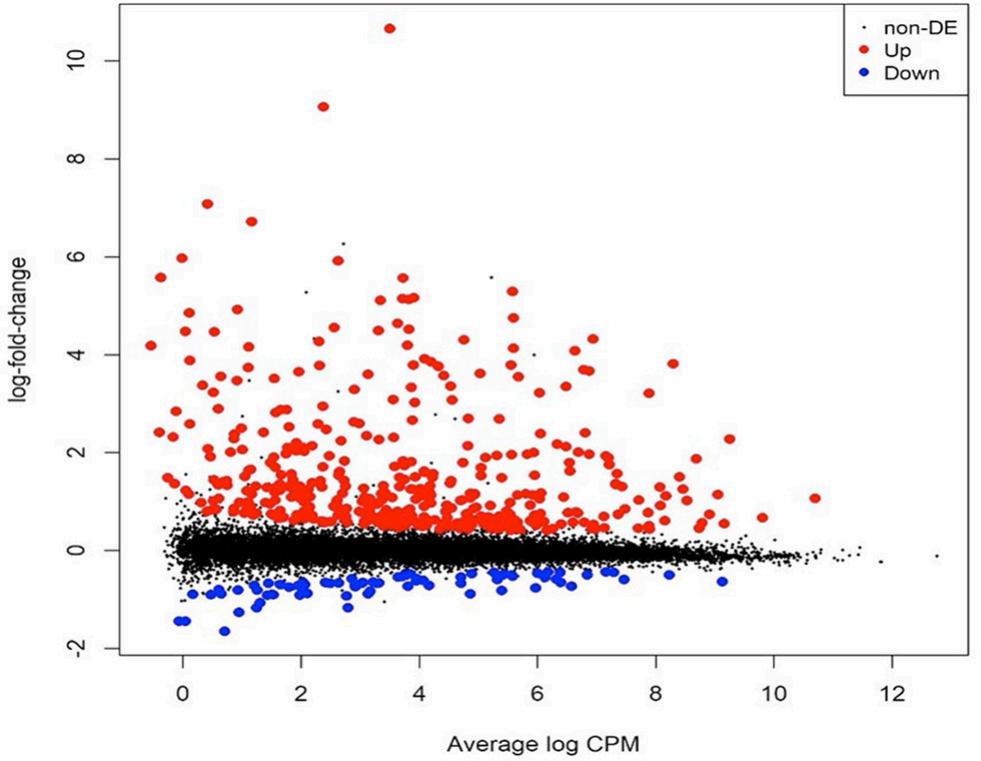
Mean-difference plot showing the log_2_ fold change (FC) and average abundance of each transcript. Significantly up- and down-regulated, differentially expressed genes based on log_2_ FC are highlighted in red and blue, respectively. RNA samples were obtained and libraries constructed from two independent experiments. The first experiment consisted of two libraries (one replicate per treatment) and the second experiment consisted of four libraries (two replicates per treatment), for a total of three biological replicates per treatment (i.e., with or without safener).

To identify gene expression changes following safener treatment in etiolated grain sorghum shoots, a total of 390 million clean reads (each 100 nucleotides long) from a total of six libraries were generated with ~70 million clean reads from each library (Table 1). Of the total reads, 99% passed quality filtering standards with 584.2 million (90.4% on average) of those reads being uniquely mapped to the sorghum genome. Approximately 5% were not mapped on a gene and 4% were multi-mapped. The raw sequences were aligned to the sorghum reference genome v. 3.1 (~90% on average) among the six libraries (Table 1). Since libraries were combined from two independent experiments to obtain replicates, PCA was performed on all six libraries to confirm the variation among libraries. Most of the variation among samples represented in PCA could be explained by the safener treatment (PC1), and the two independent sample collections (PC2) (Supplementary Figure 2). Before the analysis of DEGs between treatments, transcript reads were adjusted to remove the variance that occurred due to independent sample collections (Supplementary Figure 3). After normalization and a single pairwise comparison between control and safener-treated shoots (q-value = 0.05), 419 genes were significantly upregulated in safener-treated shoots compared to the control treatment and 73 were significantly down-regulated. The comparison between treatments identified 103 DEGs in response to safener treatment (Figure 1), whereas significantly downregulated DEGs were not detected using a threshold of log_2_ 2-fold change in expression in our studies.

**Table 1:** RNA-sequencing libraries prepared from sorghum hybrid 7431 etiolated shoots.

**Libraries**	**NumReads**	**QCfiltered**	**Unmapped**	**Mapped**	**Multimapped**	**Not.in.gene**	**In.a.gene**
WT.C.1	57671251	3264	1738123 (3.0%)	55929864 (96.9%)	1750141 (3.1%)	2822493 (5.1%)	51310389 (91.7%)
WT.C.2	62852240	4411	6025099 (9.6%)	56822730 (90.4%)	2430627 (4.3%)	3048958 (5.4%)	51294803 (90.3%)
WT.C.3	86054181	349559	2472284 (2.9%)	83232338 (96.7%)	2635497 (3.2%)	3789743 (4.6%)	76739154 (92.2%)
SA.S.1	65945347	4940	6071142 (9.2%)	59869265 (90.8%)	2459105 (4.1%)	3400576 (5.7%)	53956371 (90.1%)
SA.S.2	61534915	5320	15168564 (24.7%)	46361031 (75.3%)	3519136 (7.6%)	2718756 (5.9%)	40086035 (86.5%)
SA.S.3	88750015	384906	4265564 (4.8%)	84099545 (94.8%)	2916656 (3.5%)	4000866 (4.8%)	77111315 (91.7%)

*Three individual pots for each treatment (control or safener) were treated as biological replicates, resulting in a total of 6 libraries. Libraries were constructed from two independent experiments, where the first experiment consisted of two libraries (one replicate per treatment) and the second experiment consisted of four libraries (two replicates per treatment) for a total of three biological replicates. Two libraries from the first experiment were sequenced on a single lane that produced 100-bp single-end reads, and four libraries from the second experiment were sequenced separately on a single lane*.

**Figure 2:**
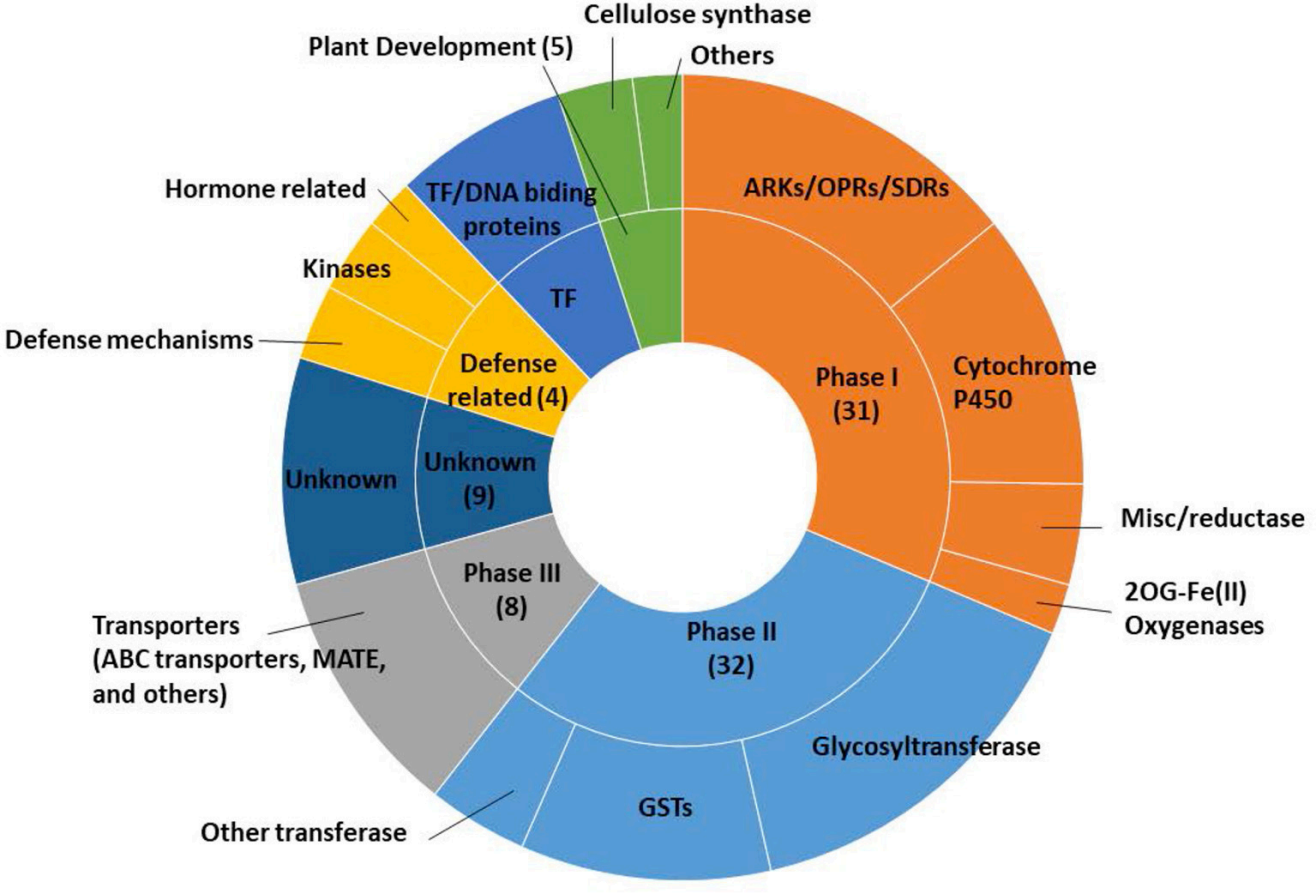
Functional categorizations related to xenobiotic detoxification of upregulated genes in etiolated sorghum shoot tissues 12 h after safener (fluxofenim) treatment.

Among the 103 differentially safener-upregulated genes, 70 transcripts are annotated as xenobiotic detoxification processes, based on *Arabidopsis* gene annotations. Those transcripts involved in detoxification processes include oxidative enzymes including P450s (11), NAD(P)-linked oxidoreductases (6), OPRs (3), short-chain dehydrogenase/reductase protein (SDR; 4) in Phase I reactions; transferases such as GSTs (11) and UGTs (17) in Phase II reactions; and transporters including ATP-binding cassette (ABC) proteins (3), multidrug resistance-associated protein (MRP; 1), and multidrug and toxic compound extrusion (MATE) efflux family protein (1) in Phase III transport processes (Supplementary Table 2 and Figure 2). The most strongly upregulated transcript (log_2_FC = 10.6) was a P450, CYP709B (Sobic.003G156200), whereas the most significantly up-regulated transcript (FDR = 1.69E−11) was an NADP-dependent oxidoreductase (Sobic.005G082600). Several upregulated genes, which were not categorized by general xenobiotic detoxification based on previous research (Baerson et al., 2005; Riechers et al., 2010; Behringer et al., 2011; Skipsey et al., 2011), may be related to phytohormone-mediated defense signaling pathways. Two transcripts encoding kinase proteins were identified including calcium-dependent protein kinases (Sobic.008G015600) and leucine-rich repeat transmembrane protein kinases (Sobic.003G402100), and one transcript encoding pathogen-related thaumatin superfamily protein (Sobic.005G226500) was identified.

The most abundant safener-upregulated transcripts (17 out of 130 genes) encode UGTs, an enzyme family that transfers glucose to diverse endogenous and xenobiotic substrates (Bowles et al., 2006; Osmani et al., 2008). Of these 17 upregulated UGTs, 10 belong to the D or L classes (five each) and 4 belong to E class. In previous research, UGTs were also the most highly represented family of upregulated genes in *Arabidopsis* following treatment with the allelochemical benzoxazolin-2(3H)-one (BOA, 11 different members; Baerson et al., 2005). In addition, the majority of safener-inducible genes encoding UGTs in rice and *Arabidopsis* in prior studies belonged to the D and L classes (Gandia-Herrero et al., 2008; Brazier-Hicks et al., 2018), and 44 *Arabidopsis* UGTs in the D, E, and L groups were responsive to 2,4,5-trichlorophenol (Brazier-Hicks et al., 2007a,b). Several of the most upregulated UGTs by the safener fenclorim in rice were also inducible by different chemical treatments such as SA, methyl jasmonate, phytoprostane-A_1_, sulfometuron-methyl, and/or pacloburtrazole (Brazier-Hicks et al., 2018).

Compartmentalization of xenobiotic conjugates in vacuoles requires transporter proteins, which are inducible by safener and xenobiotics in maize, wheat, *Arabidopsis*, and *Populus* (Gaillard et al., 1994; Rishi et al., 2004; Zhang et al., 2007; Behringer et al., 2011). Consistent with previous studies, transcripts encoding transporter-like proteins were induced by fluxofenim in our research, including four ABC transporter family proteins (Sobic.003G215800, Sobic.003G216232, Sobic.003G216166, Sobic.003G216166, and Sobic.003G267700), a MRP (Sobic.010G169000), and a MATE-type transporter (Sobic.001G185400) (Supplementary Table 2). Interestingly, the *Arabidopsis* homolog of the ABC transporter (Sobic.003G215800; AT1G15520) was also induced by BOA (Baerson et al., 2005) and by numerous other biotic and abiotic stresses (van den Brule and Smart, 2002; Campbell et al., 2003).

Transcripts possibly related to defense mechanisms other than genes encoding kinases are transcription factors (TFs) or gene-regulator proteins:ethylene-responsive element binding factor (Sobic.003G297600) and VQ motif-containing protein (Sobic.004G058000). VQ motif-containing protein is a TF known to interact with WRKY TFs, which are involved in jasmonate-dependent defense pathways (Schaller and Stintzi, 2009; Phukan et al., 2016). Interestingly, three transcripts encoding cellulose synthase-like proteins (Sobic.002G237900, Sobic.003G442500, and Sobic.006G080800) from different genetic loci were upregulated by fluxofenim, which implies the safener-mediated signaling pathway may be associated with cell development or defense processes through strengthening the cell wall and apoplast (Supplementary Table 2).

### GO and KEGG Enrichment Analysis of Sorghum DEGs Upregulated by Safener

DEGs were further compared with annotated genes in the sorghum genome. A singular enrichment analysis (SEA) was carried out with AgriGO software on the 103 upregulated genes. DEGs were assigned to different GO domains and ~26% of expressed transcripts were not assigned to any of the categories. To streamline the analysis, upregulated transcripts were categorized by cellular, metabolic processes and molecular function (Supplementary Figure 4). The significantly enriched GO categories included “oxidoreductase activity” (*P* = 0.02), and “transferase activity” (transferring hexosyl groups, *P* = 1.8e^−10^) among safener-induced genes compared with the reference genome (Supplementary Figure 4). Genes categorized by transferase and oxidoreductase activities include UGTs and NADP-dependent oxidoreductases, SDRs, and OPRs, respectively. With respect to cellular processes, genes involved in oxidation/reduction were significantly enriched (Supplementary Figure 4) while in molecular function analysis the most highly enriched genes are involved with detoxification processes.

**Figure 3:**
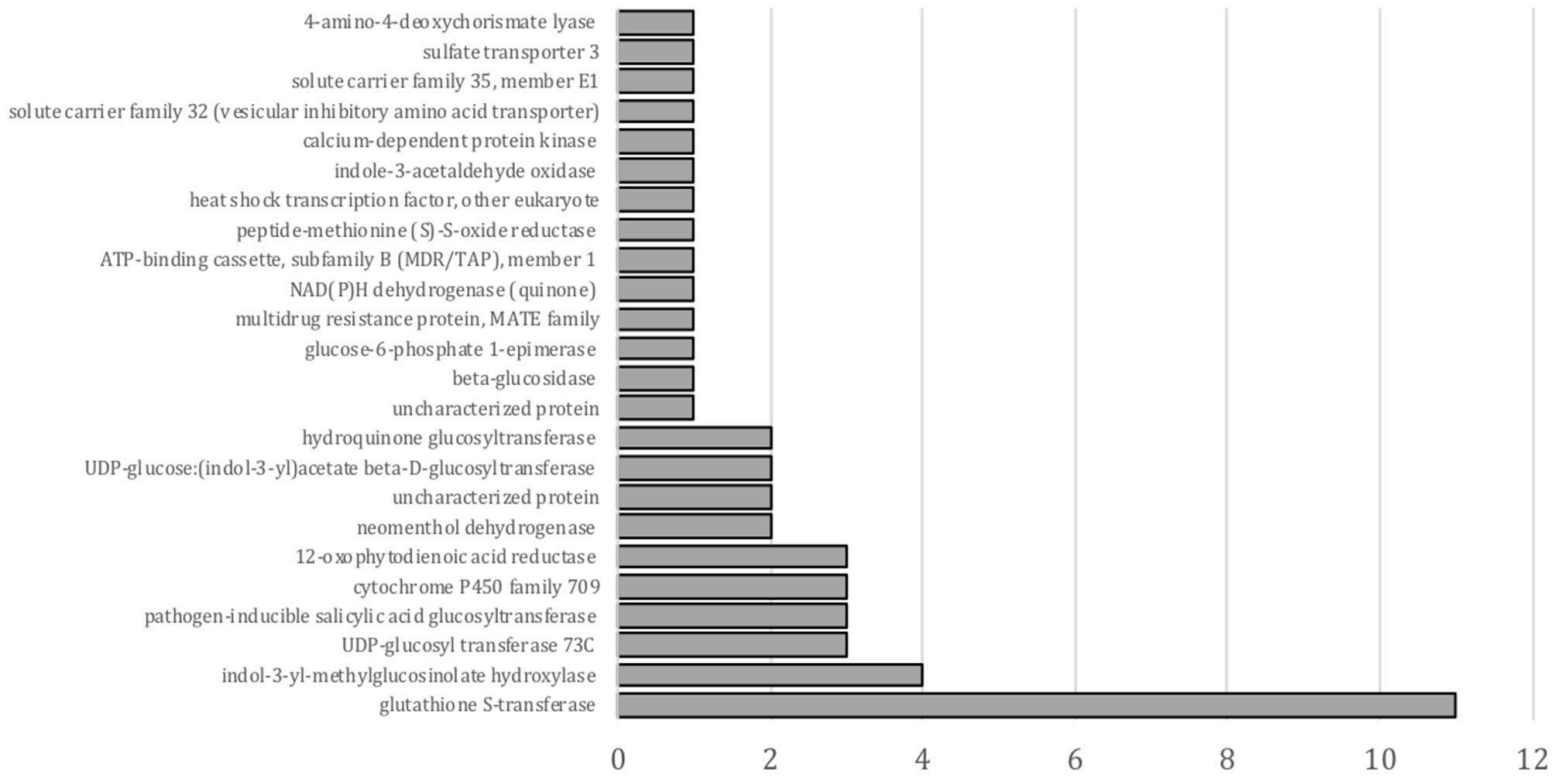
KEGG enrichment test for genes upregulated by fluxofenim treatment. The X-axis represents gene numbers.

A KEGG enrichment analysis of DEGs in a pairwise comparison of nontreated with safener- treated tissue was also conducted, although only 49 transcripts were annotated in this analysis. As shown in Figure 3, “glutathione *S*-transferase” was the most significantly enriched in upregulated transcripts by fluxofenim treatment. While transcripts related to detoxification mechanisms are enriched in the KEGG analysis as GO annotations, the analysis leads to other possible defense-signal transduction or biosynthetic secondary metabolite pathways induced by safeners (Figure 3). For example, the most enriched and upregulated glycosyltransferases (17 transcripts) were also annotated in different KEGG orthologies: “UGT73C (K13496),” “pathogen-inducible SA glucosyltransferase (K13691),” “hydroquinone glucosyltransferase (K08237),” and “UDP-glucose:(indol-3-yl) acetate beta-D-glucosyltransferase (K13692)”. This suggests that some glycosyltransferases induced by phytohormones (such as auxin and SA) may also participate in safener-regulated signaling pathways for induction of plant defense mechanisms.

### Genome-Wide Association Mapping Identifies Potential Candidate Genes Involved in Seedling Safener Response

**Figure 4:**
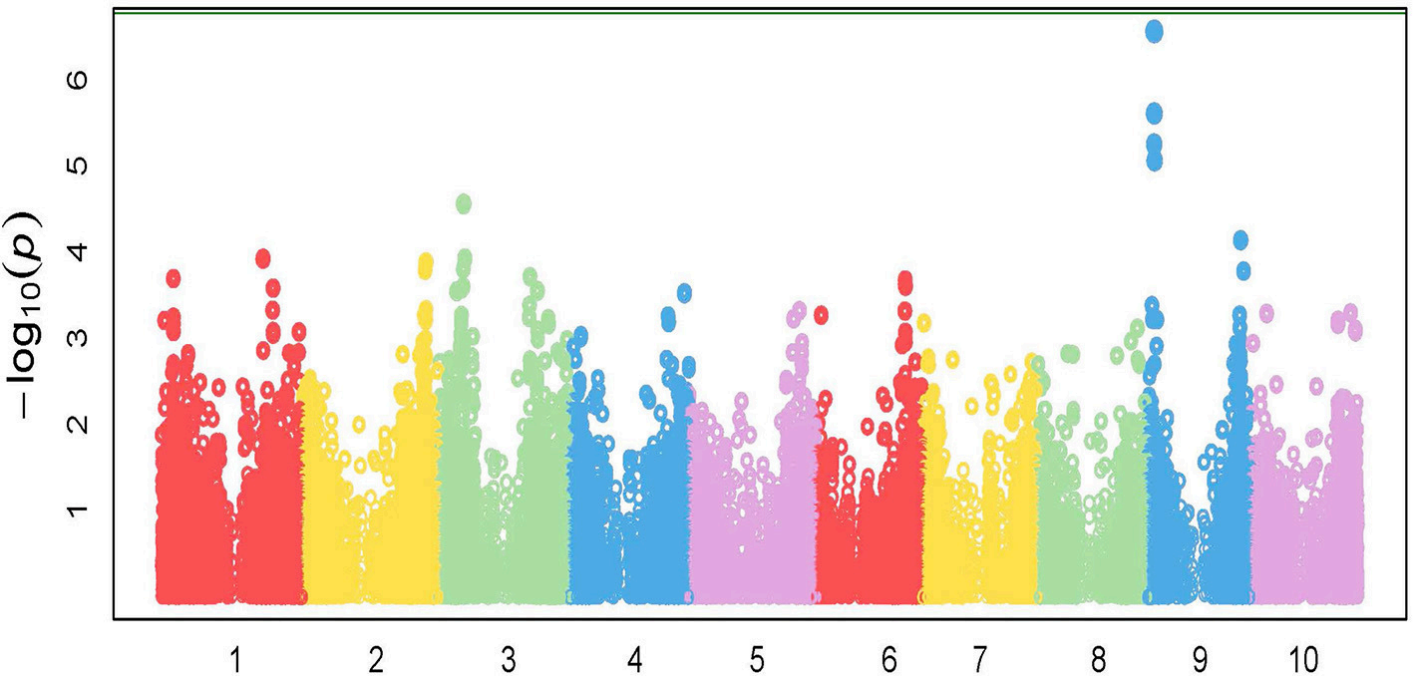
Manhattan plot of the marker-trait associations for plant fresh weight (g pot^−1^) for the safener-induced response phenotype (safener-treated vs. safener-untreated in the presence of herbicide) of grain sorghum across the sorghum reference genome (BTx623). The green horizontal line at the top of the figure represents a Bonferroni correction with α = 0.01.

**Figure 5:**
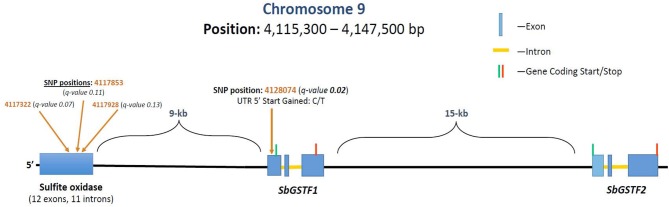
Grain sorghum safener-response associated SNPs in a 32-kb region of chromosome 9, including two tandemly duplicated *SbGSTs* and a sulfite oxidase gene. Three independent trials replicated in time (i.e., biological replicates) were conducted using a different randomization scheme in each trial. Linkage disequilibrium decays, on average, within 10–30 kb in the sorghum genome (Wang et al., 2013).

GWAS analysis was performed on the growth parameter “safener-induced response” (safener-treated *vs*. safener-untreated in the presence of herbicide) of seedling fresh weights from 761 diverse sorghum inbreds. One cluster of four SNPs was located on chromosome 9, each with q-values of < 0.13. Three SNPs fell within a putative sulfite oxidase gene (Hänsch and Mendel, 2005; Brychkova et al., 2013), but the only significant SNP (q-value = 0.016) was located at 4128074-bp on chromosome 9 within the 5′ untranslated region of a phi-class *SbGST* gene (Sobic.009G043600) and about 15-kb from a second phi-class *SbGST* gene (Sobic.009G043700) (Figures 4, 5). The two *SbGSTs* were renamed *SbGSTF1* and *SbGSTF2*, respectively, according to the proposed nomenclature system for plant GSTs (Edwards et al., 2000; Pearson, 2005). These three genes are located within a linkage disequilibrium block in the sorghum genome, which on average spans 10–30 kb (Wang et al., 2013). The minor allele of the SNP at 4128074-bp, present in 11% of inbreds tested, was associated with a reduction in safener-induced response.

In order to validate the significant GWAS-predicted SNP and associated *SbGST* genes, expression analyses were conducted using RT-qPCR and reference genes verified from among several tested previously in various sorghum tissues and organs in inbred BTx623, which contains the major allele. Inbred BTx623 has a sequenced genome (Paterson et al., 2009) and is commonly used for molecular-genetic sorghum research (Jiao et al., 2016), and was therefore utilized instead of commercial hybrid 7431 since gene-specific primers could be designed by directly analyzing genes within the publicly available genome (phytozome.jgi.doe.gov).

### Expression Analysis of Candidate *SbGSTs* Identified Through GWAS

Prior to examining the expression of the two *SbGST* genes, expression profiles of eight candidate reference genes (listed in Supplementary Table 1) were tested for stable expression in etiolated shoot tissues because their expression had only been examined previously in sorghum leaves in response to abiotic or biotic stresses (Zhang et al., 2013; Reddy et al., 2016). The primers tested amplified each candidate reference gene except *CYP* (Supplementary Figure 5A) in etiolated shoot tissues, so this transcript was omitted from further analysis. Amplicons from successful primer pairs ranged from 117- to 172-bp. Quantification cycle (Cq) values obtained from each reaction with the seven primer pairs varied in their transcript abundance. The mean Cq value for the seven candidate reference genes ranged from 17.2 to 21.0 cycles, with most falling between 19 and 21. *GTPB* had the lowest mean Cq value of 17.2, indicating the most abundant transcript level followed by *UK* at 18.8. Overall, the majority of candidate reference genes were expressed at intermediate levels with a mean Cq value of about 20 (Supplementary Figure 5B).

Three different evaluations were performed to analyze expression stability among the seven reference genes tested. Each evaluation determined a similar ranking order for gene stability but showed subtle differences; however, *GTPB, SAND*, and *PP2A.4/EIF4a* were typically the most stable genes while *ACT1* and *UK* were the least stable. Based on the consensus among the reference gene evaluations utilized (determined with the RefFinder analysis tool) and also considering PCR amplification efficiencies and regression coefficients (Supplementary Table 1), three reference genes (*SAND, GTPB*, and *EIF4a*) were selected as the most suitable, stably expressed candidate reference genes for etiolated sorghum seedling shoot tissues (Supplementary Figure 6). Furthermore, consistent and stable expression of the three references genes was also confirmed in RNA-seq reads from hybrid sorghum 7431 shoots, with and without safener treatment (Supplementary Figure 7; reads are reported as raw CPM).

**Figure 6:**
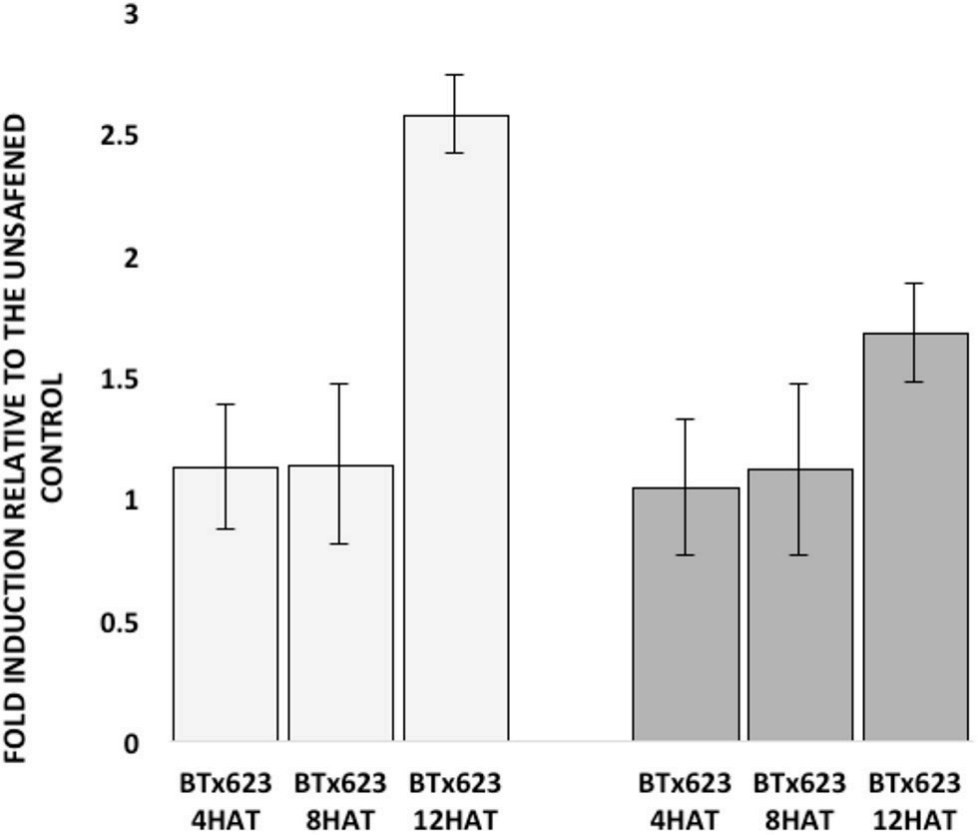
Fold induction of sorghum glutathione *S*-transferase (*GST*) genes, previously identified by GWAS, at three different hours after treatment (HAT) (**Left**, open bars). Fold induction of the *SbGSTF1* gene relative to the unsafened control for each sample at each time point. (**Right**, solid bars) Fold induction of the *SbGSTF2* gene relative to the unsafened control for each sample at each time point. Fold induction for each gene at each time point was calculated by the 2^−ΔΔCt^ method. Data represent the combined results from three independent experiments (i.e., biological replicates) with three technical replicates per sample.

Due to the high nucleotide sequence identity (81%) in the coding region between the two *SbGSTs* identified by GWAS, primer specificity was tested prior to examining their expression. Each GSP set designed for *SbGSTF1* and *SbGSTF2* only amplified products of 245-bp and 159-bp, respectively, from their specific, synthetic DNA templates (Supplementary Figure 8). Expression of the two *SbGST* genes was also tested at three different time points following fluxofenim treatment. Fold-induction levels of both *SbGSTF1* and *SbGSTF2* (relative to the controls) increased as time increased, ranging from 1-fold at 4 h after treatment (HAT) to 3-fold at 12 HAT (Figure 6). A difference in the magnitude of fold induction in response to fluxofenim was noted between the two *SbGST* genes, where *SbGSTF1* was induced at higher levels during the time course (1.1–2.6 fold) compared to *SbGSTF2* (1–1.6-fold), indicating that *SbGSTF1* is initially more responsive to fluxofenim treatment than *SbGSTF2* in this tissue. In accord with this finding, safener-induced expression of these two *SbGSTs* in hybrid 7431 in the RNAseq experiment (12 HAT; Supplementary Figure 9) is consistent with their expression in inbred BTx623 using RT-qPCR described above (Figure 6). These results imply that induction of *SbGSTF1 and SbGSTF2* expression may play a role in determining phenotypic responses to herbicide plus safener applications and is in accord with the GWAS results (Figures 4, 5). However, additional functional analyses are needed to further explore this hypothesis.

## Discussion

### Transcriptional Reprogramming by Safener in Etiolated Grain Sorghum Shoots

Although mechanisms of safener action have been investigated at the transcriptome level in several prior studies with model dicots (Rishi et al., 2004; Behringer et al., 2011), the precise underlying molecular basis of how safeners regulate defense and detoxification mechanisms is not fully understood. In the current study, RNAseq and GWAS approaches allowed for identification of new candidate genes involved with the safener response in sorghum and yielded new insights about possible safener-mediated signaling mechanisms. For example, all DEGs consisted of upregulated transcripts (Figure 1), indicating that the safener response at 12 HAT appears to involve transcriptional activation and not repression. Importantly, among the 103 differentially upregulated genes, 70 genes homologous to *Arabidopsis* gene annotations potentially encode important detoxification enzymes including oxidative enzymes, transferases and vacuolar transporter proteins (Figure 2 and Supplementary Table 2). This finding is consistent with previous studies showing induction of individual components of the cellular detoxification machinery by safeners in dicots, including *Arabidopsis* and *Populus* (Rishi et al., 2004; Smith et al., 2004; DeRidder and Goldsbrough, 2006; Behringer et al., 2011). Similar findings were also reported for the diploid wheat *Aegilops tauschii* in response to the safener cloquintocet-mexyl (Zhang et al., 2007). Some of these enzymes are not only upregulated by safeners but are also inducible by various xenobiotics, including the allelochemical BOA (Baerson et al., 2005) and environmental pollutant 3,4-dichloroaniline (Loutre et al., 2003).

The majority of safener-inducible plant GSTs identified in previous research (Riechers et al., 1997; Zhang et al., 2007; Dixon et al., 2009; Brazier-Hicks et al., 2018) belong to the tau class. Similarly, eight of the 11 transcripts encoding GSTs identified in this study belong to the tau class (Supplementary Table 2). In addition to eight tau-class GSTs, one lambda-class and two phi-class GSTs were upregulated by fluxofenim. Interestingly, it is still not understood why safeners upregulate *GSTs* and other defense/detoxification genes in dicot plants without eliciting a phenotypic response; i.e., confer protection from herbicide injury (DeRidder et al., 2002; Riechers and Green, 2017). For example, *AtGSTU19* expression was most abundant in the roots of *Arabidopsis* seedlings rather than shoots (DeRidder and Goldsbrough, 2006). Differential gene expression among seedling tissues may result from differing anatomy between monocot and dicot seedlings during seedling development, which might partially explain why dicots are not protected from soil-applied herbicide injury by safener treatment. Taken together, these findings suggest that detoxification mechanisms regulated by safeners require not only certain subclasses of GSTs with the appropriate substrate specificities, but also require the proper subcellular location and tissue distribution of GST expression (Riechers et al., 2003, 2010).

Detoxification enzymes are often upregulated by oxylipins derived from fatty acid oxidation (Loeffler et al., 2005; Mueller et al., 2008; Mueller and Berger, 2009), thus leading to the hypothesis that safeners may in part utilize an oxylipin-mediated signaling pathway to induce detoxification enzymes in cereal crops (Riechers et al., 2010; Skipsey et al., 2011). Common genes, such as *GSTs, P450s, UGTs*, and *OPRs*, are induced by both safeners and oxylipins in *Arabidopsis* (Taki et al., 2005; Mueller et al., 2008; Riechers et al., 2010; Behringer et al., 2011). Consistent with previous findings, 24 safener-upregulated transcripts in this study (*P450s, GSTs, UGTs, OPR2*, and an ABC transporter; Supplementary Table 2) were also reported as inducible by oxylipin treatment in prior studies. However, a recent study examining possible phenotypic effects provided by oxylipin treatments (OPDA and phytoprostane-A_1_) toward herbicide safening did not show complete protection in rice, despite the induction of *GST* expression (Brazier-Hicks et al., 2018). Collectively, these results imply that oxylipin-signaling pathways may be a secondary pathway safeners utilize to enhance seedling protection from herbicide injury, or that additional, essential signaling pathways might operate in parallel.

### Biosynthesis, Metabolism, and Cellular Sequestration of Chemical Defense Compounds

The vast majority of safener-upregulated transcripts identified in our study are involved in detoxification, but several transcripts also fell within categories related to plant secondary metabolism (Figure 3). The most well-studied example of a biosynthetic pathway for secondary metabolites in grain sorghum is the chemical defense compound dhurrin, a toxic cyanogenic glycoside (Halkier and Møller, 1989; Tako and Rook, 2012; Darbani et al., 2016; Nielsen et al., 2016; Bjarnholt et al., 2018; Yeats, 2018). Interestingly, strong similarities exist between the safener-upregulated transcripts identified in our research and the genes/proteins that participate in synthesis and catabolism of dhurrin (Halkier and Møller, 1989; Darbani et al., 2016). For example, dhurrin synthesis is catalyzed by two P450s and UGT85B1 that form a gene cluster on chromosome 1, which co-localize and are co-expressed with genes encoding *SbMATE* and an *SbGST* (Sobic.001G412700) (Tako and Rook, 2012; Blomstedt et al., 2016; Hayes et al., 2015; Darbani et al., 2016). Although several safener-upregulated transcripts identified in our study encode P450s (6), GSTs (6), UGTs (2), and a MATE protein (Sobic.001G185400) located on chromosome 1, they are not identical to those transcripts encoding dhurrin biosynthetic genes in sorghum grain (Darbani et al., 2016).

In contrast to the genes described above, fluxofenim induced the transcript encoding a specific β-glucosidase [dhurrinase, (Sobic.002G261600)] in our study that degrades dhurrin and releases hydrogen cyanide (HCN) upon tissue disruption by pathogen or insect attack (Halkier and Møller, 1989; Cicek and Esen, 1998; Morant et al., 2008; Nielsen et al., 2008; Gleadow and Møller, 2014). The rate of dhurrin metabolism as compared to HCN concentration exceeds synthesis in the later stages of seedling growth. However, dhurrin concentration decreased (with a concomitant lack of HCN production) during later stages of grain development, suggesting that dhurrin is turned over and recycled to avoid auto-toxicity (Busk and Møller, 2002; Gleadow and Møller, 2014; Nielsen et al., 2016). In relation to a previous profiling study of dhurrin concentrations and transcriptomes during sorghum grain development (Nielsen et al., 2016), the majority (10 out of 17) of safener-upregulated transcripts encoding UGTs in our research are similar to those induced after dhurrin dissipates (Nielsen et al., 2016). Moreover, eight of 11 safener-induced *GSTs* in our study are also expressed in sorghum grain during later developmental stages when dhurrin concentrations decline. A more recent study revealed that two specific lambda-class GSTs (SbGSTL1 and SbGSTL1; transcripts Sobic.002G421200 and Sobic.009G033200) participate in the dhurrin recycling pathway via reductive cleavage of a dhurrin-derived glutathione conjugate, *p*-hydroxyphenyl(*S*-glutathione)acetonitrile (Bjarnholt et al., 2018), thereby preventing HCN accumulation. Fluxofenim predominantly induced several tau-class GSTs, but additionally a similar but distinct lambda-class GSTL (Sobic.001G412700) was induced by safener treatment. This transcript encodes SbGSTL3, which does not convert *p*-hydroxyphenyl(*S*-glutathione)acetonitrile to *p*-hydroxyphenyl acetonitrile (Bjarnholt et al., 2018), but shares high sequence identity with the maize safener-inducible In2-1 protein (Hershey and Stoner, 1991). While the majority of dhurrin accumulates in young sorghum seedlings (mainly in coleoptiles) at 48 h after germination (Halkier and Møller, 1989; Bjarnholt et al., 2018), the shoot RNAseq libraries in our transcriptome analysis were prepared from sorghum shoots collected at a total of 54 h after germination (42 h after germination then 12 h after fluxofenim). This suggests that the safener-mediated signaling pathway and the dhurrin biosynthetic pathway may share a common regulator, such as a TF and/or signaling molecules, to promote similar gene and protein expression profiles during early seedling development in sorghum. It is also possible that dhurrin and fluxofenim share common detoxification enzymes involved in their own metabolism in sorghum shoots, based on similarities between their chemical structures (Supplementary Figure 10). In support of this theory, previous reports indicated that GSTs can metabolize both safeners and the herbicides from which they protect in cereal crops (reviewed by Riechers et al., 2010).

It has not been determined if the safener-upregulated transcripts on chromosome 1 are transcriptionally co-regulated. However, four upregulated transcripts encoding P450s on chromosome 1 (Sobic.001G082200, Sobic.001G082300, Sobic.001G082400, and Sobic.001G082500) are clustered, possibly within the physical range for gene co-regulation along with a *MATE* and several *GSTs* (e.g., Sobic.001G185400, Sobic.001G065900, and Sobic.001G318700) (Reimegård et al., 2017; Soler-Oliva et al., 2017). Clustering of non-homologous genes involved in the same biological process, such as dhurrin biosynthetic genes, may be an adaptive trait that enables coordinated regulation and inheritance while maintaining a functional metabolic pathway (Tako and Rook, 2012; Darbani et al., 2016) and suggests a common system in plants to efficiently avoid auto-toxicity following chemical defense synthesis (Busk and Møller, 2002). For example, P450s determine the specificity of xenobiotic detoxification by recognizing distinct substrates (Siminszky, 2006; Mizutani and Ohta, 2010) while UGTs and MATEs coordinately function in the conjugation and transport of secondary compounds from the cytosol to the vacuole. These coordinated cellular processes likely minimize the risk of auto-toxicity, as is the case with many constitutive and inducible plant defense compounds (Mithöfer and Boland, 2012; Gleadow and Møller, 2014) and secondary metabolites (Gierl and Frey, 2001; Frey et al., 2009; Winzer et al., 2012).

### Genome-Wide Association Studies

A 32-kb interval on chromosome 9, which comprises *ca*. one linkage disequilibrium block within the sorghum genome (Wang et al., 2013), contains a sulfite oxidase gene and two sorghum phi-class *GST* genes (*SbGSTF1* and *SbGSTF2*), of which *SbGSTF1* is strongly associated with variation in safener-induced response phenotypes. Five main GST subclasses exist in plants, of which tau- and phi-class GSTs are the most abundant (Labrou et al., 2015). A total of 99 *GSTs* have been identified in the sorghum genome with a distribution of 64% tau-class and 22% phi-class genes (Chi et al., 2011), which is typical of cultivated cereal crops, and frequently occur as tandem gene duplications. Both GST classes are closely associated with plant stress responses and their expression is highly responsive to external stimuli such as herbicides (Cummins et al., 2011). GSH is required as a co-substrate for GST-catalyzed conjugation of xenobiotics in plants (Farago and Brunold, 1990; Farago et al., 1994). Plant sulfite oxidases are peroxisomal enzymes involved with maintaining leaf sulfite homeostasis (Brychkova et al., 2013), protecting cells from sulfitolysis (Hänsch and Mendel, 2005), and detoxifying sulfur dioxide (Brychkova et al., 2007) but are not involved with primary sulfate assimilation in plants (Hänsch and Mendel, 2005), so their possible role in alleviating herbicide-induced phytotoxicity is unclear. The presence of safener-upregulated *SbGSTF1* and *SbGSTF2* and lack of sulfite oxidase transcripts in the RNAseq experiment (Supplementary Table 2), however, strongly implicate the tandem *SbGSTs* as part of a major QTL associated with the safener-induced phenotype in grain sorghum.

Using the sorghum reference genome, the deduced amino acid sequences of SbGSTF1 and SbGSTF2 were compared to the N-terminal amino acid sequences of several safener-upregulated phi-class GST isozymes purified and biochemically characterized previously and before the sorghum genome was publicly available (Gronwald and Plaisance, 1998). However, based on alignments with these partial N-terminal sequences, SbGSTF1 and SbGSTF2 are different safener-responsive phi-class SbGSTs. However, the coding region of the deduced *SbGSTF2* cDNA is 81% identical with the coding region of the *SbGSTF1* cDNA (data not shown). As a result, we hypothesize that *SbGSTF2* is most likely a paralog of *SbGST1* (Lynch, 2013) resulting from a relatively old tandem duplication event (Chi et al., 2011). Fluxofenim-induced *SbGSTF1* and *SbGSTF2* expression levels in inbred BTx623 shoots increased with time (Figure 6), consistent with the theory that inducible detoxification mechanisms are preferred over constitutively expressed mechanisms in response to variable, unpredictable stress exposure (Züst and Agrawal, 2017). The difference in the scale of gene expression in response to fluxofenim between *SbGSTF1* and *SbGSTF2* may reflect functional divergence of these paralogous genes (Lynch, 2013). For example, as a result of this duplication event one of the *SbGSTs* may have acquired additional function(s) that the other lacks, or could have lost by accumulating degenerative mutations (Innan and Kondrashov, 2010). Although our GWAS study identified two specific phi-class *SbGSTs* associated with safener-induced responses, further studies such as analyzing substrate specificity and protein localization in sorghum shoot tissues and cells, before and after safener treatment, are required to fully understand the function of these phi-class GSTs in detoxification and/or inducible defense processes.

By understanding which genes to target via cis-genic manipulation (i.e., CRISPR-Cas9) or conventional plant breeding, an increase of certain enzyme activities encoded by these genes could result in the development of grain sorghum lines that are tolerant to a wide range of xenobiotics, as well as abiotic and biotic stresses. Since increasing weed pressure and development of herbicide- resistant weed populations are two of the biggest problems facing agriculture today (Yu and Powles, 2014), it is imperative to develop more effective, herbicide-resistant and stress-tolerant crop varieties. Chemical manipulation of mechanisms that regulate metabolic detoxification of natural and synthetic toxic compounds is of great agricultural interest, such as safener-regulated enhancement of herbicide tolerance and the greater herbicide selectivity margin between cereal crops and target weeds (Riechers and Green, 2017). This is a particularly important trait in cultivated grain sorghum where wild, weedy relatives preclude the development of genetically modified varieties due to high risk of gene flow (Ohadi et al., 2017).

Overall, our integrated approach for identification of safener-responsive genes and transcripts has provided new information about the safener-regulated signaling pathway in sorghum shoots, as well as assist in defining the precise mechanism by which safeners upregulate xenobiotic detoxification pathways in cereal crops. Based on our global transcriptome analysis, we now hypothesize that safeners utilize the same or similar biosynthetic and recycling pathways typically used for chemical defense compounds to also enhance herbicide tolerance, in addition to phytohormone- or oxylipin-mediated signaling pathways. Future studies by our research group will further investigate safener-mediated signaling pathways and detoxification mechanisms in dissected coleoptiles from safener-treated, etiolated sorghum shoots to comprehensively understand cell- and tissue-specific gene expression patterns.

## Author Contributions

DR and PB conceptualized and designed the work. YB, BJ, and SM acquired and analyzed the RNA-seq transcriptome data. LG and PB conducted and analyzed the genome-wide association studies. LG and KL performed RT-qPCR, validated candidate reference genes, and analyzed *SbGST* expression. YB, LG, PB, and DR wrote the manuscript. All authors revised and approved the final version to be published and agree to be accountable for all aspects of the work in ensuring that questions related to the accuracy or integrity of any part of the work are appropriately investigated and resolved.

### Conflict of Interest Statement

The authors declare that the research was conducted in the absence of any commercial or financial relationships that could be construed as a potential conflict of interest.

## Supplementary Material

Supplementary Table 1 Primer sequences used for quantitative RT-PCR analysis.

Supplementary Table 2 Classification of safener-induced transcripts based on detoxification pathways.

Supplementary Figure 1 Safener and herbicide responses in grain sorghum hybrid 7431 seedlings two weeks after treatment.

Supplementary Figure 2 Genome-wide variation in gene expression across six libraries.

Supplementary Figure 3 Plots of the quarter-root QL dispersion against the average abundance of each gene before **(A)** and after **(B)** adjusting sequencing batches.

Supplementary Figure 4 Gene ontology analysis of safener-upregulated genes for molecular **(A)** and cellular processes **(B)**.

Supplementary Figure 5 Confirmation of amplicon size and primer specificity for eight candidate reference genes **(A)** and expression levels of the final seven candidate reference genes **(B)**.

Supplementary Figure 6 Expression stability and ranking of the seven candidate reference genes calculated by three different evaluation methods.

Supplementary Figure 7 Raw CPM reads of three selected references genes *(GTPB, EIF4a, and SAND)* by RNAseq analysis at 12 h after treatment with 20 μM fluxofenim in sorghum hybrid 7431 shoots.

Supplementary Figure 8 Specificity of glutathione *S*-transferase *(SbGSTF1 and SbGSTF2)* gene primers.

Supplementary Figure 9 Raw CPM reads from the two tandem *SbGSTs* via RNA-seq analysis at 12 h after treatment with 20 μM fluxofenim in sorghum hybrid 7431 shoots.

Supplementary Figure 10 Chemical structures of the sorghum safener fluxofenim (**A**; an oxime ether) and the cyanogenic glycoside dhurrin **(B)**.
