# Supplementary material for: Transcriptome Profiling and Genome-Wide Association Studies Reveal GSTs and Other Defense Genes Involved in Multiple Signaling Pathways Induced by Herbicide Safener in Grain Sorghum
Source: Front Plant Sci. 2019 Mar 8;10:192. doi: 10.3389/fpls.2019.00192 (PMC6418823; doi:10.3389/fpls.2019.00192)
Supplement: Supplementary Table 1 — Primer sequences used for quantitative RT-PCR analysis. [file Table_1.pdf]

Supplementary Table 1 | Primer sequences used for quantitative RT-PCR analysis.

Gene Symbol	Primers sequences (5'-3') (forward/reverse)	Amplicon length (bp)	T_m (°C)	PCR Efficiency (%)	Regression coefficient (R^2)
**REFERENCE GENES**					
PP2A.1	ATGCGGTGATATTCATGGACAA<br>GAGTAGTAACCACGGTCAACATAA	117	62	106.8	0.988
PP2A.4	GTGGCCTCTCTCCGTCATTG<br>TGTAACCTGCTCCTCTTGGTG	153	61.5	107.7	0.997
GTPB	ACACTGCTGGGCAAGAGAAG<br>TTACCGCAGAGGACAATGGG	172	62	105.8	0.999
UK	CACAGTTGTGGATGCCGCT<br>TGTGCCCTTTCCACTTCCAG	118	63	108.2	0.976
EIF4a	CTGTCCGTGAGGACCAAAGG<br>CTTGAATCCACGGGAGAGCA	161	63.5	102.2	0.998
CYP	TTGTTCTCGCACCCTGATCG<br>GATGTCGGTGGGGGAAAAGG	137	65.5	NA*	NA*
SAND	GAAAGCGCCCTTTCCTGGT<br>GACAAACCCAGCCCCTCATA	151	62	106	0.996
ACT1	CTAGCAGCATGAAGATCAAGGTG<br>GCCAGACTCGTCGTACTCAG	134	60	125.6	0.992
**TARGET GENES**					
SbGSTF1	ACGTTCTCCGCAAGCACAA<br>GTACTTGCTCTCGCTCAGCC	245	62	98.4	0.997
SbGSTF2	GAAGCTCAAGAAGGTGCTGG<br>ACATGAGGGAGTGCCTTGAC	159	62.4	104.5	0.998

**CYP* reference gene primers did not produce a PCR product. The *CYP* gene was not used in subsequent experiments.

Supplementary Table 2 | Classification of safener-induced transcripts based on detoxification pathways. Results for transcriptional regulation by each compound listed in this Table are derived from the following original references: 12-oxophytodienoic acid (OPDA) and phytoprostane-A_1 (PPA_1) (Taki et al., 2005; Mueller et al., 2008; Sham et al., 2014); safener (Behringer et al., 2011); brassinosteroids (BR) and auxin (IAA) (Goda et al., 2004); and pathogens, jasmonates (JA) or ethylene (ET) (Libault et al., 2007; Frerigmann et al., 2014; Zhao et al., 2007).

**Locus Name**	***Arabidopsis* ID**	**Arabi. Define**	**Log_2 FC**	**FDR**	**Regulated by[1]**	**Notes on Function[2]**
**Phase I Metabolism**						
Cytochromes P450						
Sobic.001G082200	AT4G37320	Cytochrome P450, CYP81D5	2.2	1.4E-07		
Sobic.001G082300	AT4G37320	Cytochrome P450, CYP81D5	2.4	0.0481		
Sobic.001G082400	AT4G37320	Cytochrome P450, CYP81D5	2.3	0.0070		
Sobic.001G082500	AT2G23190	Cytochrome P450, CYP81D7	3.3	0.0123		
Sobic.007G128000	AT4G37330	Cytochrome P450, CYP81D4	4.9	1.27E-07	Safener+	
Sobic.001G195100	AT1G64940	Cytochrome P450, CYP87A6	3.4	5.98E-05		
Sobic.001G195200	AT1G64940	Cytochrome P450, CYP87A6	3.8	0.0147		
Sobic.002G388900	AT2G46950	Cytochrome P450, CYP709B2	4.8	0.0049	Safener+	
Sobic.002G389000	AT2G46950	Cytochrome P450, CYP709B2	5.1	6.20E-06		
Sobic.003G156200	AT2G46950	Cytochrome P450, CYP709B2	10.6	1.69E-08		
Sobic.003G228100	AT3G14690	Cytochrome P450, CYP72A5	3.2	4.84E-08	Safener+, OPDA+, PPA_1+	
Oxidoreductase						
Sobic.008G112000	AT1G06620	2-oxoglutarate (2OG) and Fe(II)-dependent oxygenase superfamily protein	2.3	1.21E-05	Pathogen^	Synthesis of benzoxazinoids
Sobic.001G062300	AT3G43600	Aldehyde oxidase 2	2.1	1.67E-08		Involved in ABA biosynthesis

AKRs/OPR/SDRs						
Sobic.001G231400	AT4G13180	Short-chain dehydrogenase/reductase (SDR) family protein	3.8	3.55E-09	Safener+	
Sobic.004G303800	AT1G01800	Short-chain dehydrogenase/reductase (SDR) family protein	3.9	0.0171	OPDA+	
Sobic.004G304000	AT1G01800	Short-chain dehydrogenase/reductase (SDR) family protein	4.5	0.0019	OPDA+	
Sobic.004G304100	AT1G01800	Short-chain dehydrogenase/reductase (SDR) family protein	3.9	3.38E-09	OPDA+	
Sobic.006G091700	AT1G76680	12-oxophytodienoate reductase 1	4.5	0.0006	Safener+, PPA_1+	
Sobic.010G084600	AT1G76690	12-oxophytodienoate reductase 2	4.2	2.27E-06	Safener+, OPDA+, PPA_1+	Detoxification of benzoxazolinone
Sobic.010G084700	AT1G76690	12-oxophytodienoate reductase 2	3.9	0.0284	Safener+, OPDA+, PPA_1+	Detoxification of benzoxazolinone
Sobic.002G074400	AT5G54500	Flavodoxin-like quinone reductase 1	3.1	0.0118	Safener+, IAA^ Pathogen^	Detoxification of benzoxazolinone
Sobic.001G500200	AT5G05320	FAD/NAD(P)-binding oxidoreductase family protein	5.3	0.0038	JA+, ET+	
Sobic.005G008201	AT1G60730	NAD(P)-linked oxidoreductase superfamily protein	4.9	4.11E-09	OPDA+, Pathogen^	Detoxification of benzoxazolinone
Sobic.005G008301	AT1G60710	NAD(P)-linked oxidoreductase superfamily protein	6.0	7.79E-08		Detoxification of reactive carbonyls
Sobic.010G117900	AT1G60690	NAD(P)-linked oxidoreductase superfamily protein	5.2	8.43E-08		Detoxification of reactive carbonyls
Sobic.005G082600	AT3G03080	Putative NADP-dependent oxidoreductase	5.6	1.69E-11		
Sobic.005G082700	AT3G03080	Putative NADP-dependent oxidoreductase	2.5	1.67E-08		
Misc/Reductase						
Sobic.006G123300	AT5G61640	Peptidemethionine sulfoxide reductase	2.0	0.0001		
Sobic.006G205000	AT5G57330	Galactose mutarotase-like superfamily protein	2.4	4.50E-05		

Sobic.001G004500	AT2G37210	Lysine decarboxylase family protein	2.1	0.0011		
**Phase II Metabolism**						
GSTs						
Sobic.003G425850	AT3G09270	Glutathione *S*-transferase TAU 8	5.9	2.94E-08	Safener+, OPDA+, PPA_1+	Detoxification of benzoxazolinone
Sobic.002G361100	AT1G17180	Glutathione *S*-transferase TAU 25	4.6	1.69E-11	Safener+, PPA_1+, Pathogen^	Detoxification of benzoxazolinone
Sobic.001G065900	AT1G17180	Glutathione *S*-transferase TAU 25	4.3	0.0087	Safener+, PPA_1+, Pathogen^	Detoxification of benzoxazolinone
Sobic.001G318700	AT1G10370	Glutathione *S*-transferase TAU 17	3.6	1.64E-05		
Sobic.001G319500	AT1G10370	Glutathione *S*-transferase TAU 17	4.1	0.0008	Safener+	
Sobic.001G318200	AT1G10360	Glutathione *S*-transferase TAU 18	2.9	0.0002		
Sobic.002G173200	AT1G53680	Glutathione *S*-transferase TAU 28	9.1	2.28E-09		
Sobic.001G066000	AT1G53680	Glutathione *S*-transferase TAU 28	3.6	2.65E-07	Safener+	
Sobic.009G043600	AT3G62760	Glutathione *S*-transferase Phi 13	3.8	5.48E-11		
Sobic.009G043700	AT3G62760	Glutathione *S*-transferase Phi 13	3.5	2.13E-05		
Sobic.001G412700	AT3G55040	Glutathione transferase Lambda 2	3.8	0.0143		
Glycosyltrasnferase						
Sobic.003G042900	AT4G15550	UDP-glucose:indole-3-acetate beta-D-glucosyltransferase (IAGLU)	4.1	0.0006	Safener+,	
Sobic.001G084200	AT3G02100	UDP-glucosyltransferase	2.0	9.35E-06		
Sobic.001G084400	AT3G02100	UDP-glucosyltransferase	3.1	5.95E-08		
Sobic.002G265600	AT1G05680	UDP-glycosyltransferase 74E2	4.6	0.0125	Safener+, PPA_1+,	Detoxification of benzoxazolinone
Sobic.002G265800	AT2G43820	UDP-glucosyltransferase 74F2	3.7	0.020	Safener+	Detoxification of benzoxazolinone
Sobic.002G265900	AT2G43820	UDP-glucosyltransferase 74F2	3.7	2.86E-06	Safener+	Detoxification of benzoxazolinone

Sobic.003G047500	AT2G36780	UDP-glycosyltransferase superfamily protein	4.5	4.15E-11		
Sobic.003G047600	AT2G36780	UDP-glycosyltransferase superfamily protein	7.1	3.1E-08		
Sobic.003G232900	AT2G15480	UDP-glucosyl transferase 73B5	3.2	6.30E-07	Safener+, OPDA+, Pathogen^	Detoxification of benzoxazolinone
Sobic.003G233000	AT2G15480	UDP-glucosyl transferase 73B5	3.6	2.65E-07	Safener+, OPDA+, Pathogen^	Detoxification of benzoxazolinone
Sobic.003G267900	AT3G16520	UDP-glucosyl transferase 88A1	2.1	0.0002		
Sobic.003G287500	AT3G16520	UDP-glucosyl transferase 88A1	3.7	0.0031		
Sobic.005G032200	AT1G05560	UDP-glucosyltransferase 75B1	4.6	0.01247	Safener+, PPA_1+	Detoxification of benzoxazolinone
Sobic.009G080400	AT4G01070	UDP-glycosyltransferase superfamily protein	3.7	8.43E-09	PPA+	Detoxification of benzoxazolinone
Sobic.009G205700	AT3G16520	UDP-glucosyl transferase 88A1	2.4	3.58E-06		
Sobic.010G179400	AT1G01420	UDP-glucosyl transferase 72B3	2.4	0.0151	PPA_1+, OPDA+, Pathogen ^	
Sobic.010G091100	AT2G36800	DON-glucosyltransferase 1	6.7	1.04E-08		
Other transferases						
Sobic.007G059200	AT3G29670	HXXXD-type acyl-transferase family protein	4.5	7.81E-08		
Sobic.003G260000	AT3G29670	HXXXD-type acyl-transferase family protein	3.3	0.0023		
Sobic.010G238600	AT3G29590	HXXXD-type acyl-transferase family protein	2.7	5.78E-09		
Sobic.009G088800	AT5G57850	D-aminoacid aminotransferase-like PLP-dependent enzymes	3.0	1.71E-06		
Sobic.001G397400	AT3G16910	Acyl-activating enzyme 7	2.4	0.0009	ET-	
**Phase III**						
Transporters						

Sobic.010G169000	AT3G21250	Multidrug resistance-associated protein 6	2.0	3.50E-06	Safener+, PPA_1+	Detoxification of benzoxazolinone
Sobic.003G215800	AT1G15520	ABC transporter family protein	4.3	2.55E-11	Safener+, OPDA+, PPA_1+, Pathogen^	Detoxification of benzoxazolinone
Sobic.003G216232	AT1G15520	ABC transporter family protein	3.0	0.0275	Safener+, OPDA+, PPA_1+, Pathogen^	TGA dependent
Sobic.003G216166	AT1G15520	ABC transporter family protein	2.9	0.0021	Safener+, OPDA+, PPA_1+, Pathogen^	TGA dependent
Sobic.003G267700	AT2G47000	ATP binding cassette subfamily B4	2.6	0.00212	OPDA+, PPA_1+, Pathogen^	
Sobic.001G185400	AT3G21690	MATE efflux family protein	2.5	8.40E-07	JA+	
Sobic.002G322800	AT5G64700	Nodulin MtN21 /EamA-like transporter family protein	2.5	3.15E-08		
Sobic.003G278600	AT3G51895	Sulfate transporter 3;1	2.6	1.7E-05	IAA+	
Sobic.004G325300	AT2G41190	Transmembrane amino acid transporter family protein	2.2	6.13E-06		
Sobic.003G050800	AT5G33320	Glucose-6-phosphate/phosphate translocator-related	2.3	1.47E-06		
**OTHERS**						
DNA binding proteins/TFs						
Sobic.003G297600	AT2G44840	Ethylene-responsive element binding factor 13	2.8	1.53E-07	Pathogen^, IAA+	
Sobic.001G020200	AT1G34370	C2H2 and C2HC zinc fingers superfamily protein	2.6	0.00130	OPDA+	
Sobic.003G385100	AT5G67480	BTB and TAZ domain protein 4	2.0	2.15E-07	IAA+	Proteasome-mediated ubiquitin-dependent protein catabolic process
Sobic.009G208000	AT5G45710	Winged-helix DNA-binding transcription factor family protein	2.9	6.13E-06		Member of heat stress transcription factor (Hsf) family
Sobic.004G058000	AT4G39720	VQ motif-containing protein	2.1	1.04E-06		Interacts with WRKY TF

						(JA/ET+)
Sobic.003G105800	AT3G04070	NAC domain containing protein 47	3.6	2.33E-07		Detoxification of beznoxazolinone<br>Involved in ET biosynthesis
Sobic.010G125400	AT3G13310	Chaperone DNAJ-domain superfamily protein	3.8	0.0002		Interacts with heat-shock proteins (Hsps)/chaperones
Plant Defense-Related						
Sobic.005G226500	AT2G28790	Pathogenesis-related thaumatin superfamily protein	2.1	8.56E-06	Pathogen^	
Sobic.009G009300	AT5G23960	Terpene synthase 21	4.3	8.17E-09		
Sobic.002G078700		LTPL169 - Protease inhibitor/seed storage/LTP family protein precursor, expressed	3.4	8.40E-07		
Sobic.002G078500		LTPL169 - Protease inhibitor/seed storage/LTP family protein precursor, expressed	2.7	0.0370		Detoxification of beznoxazolinone
Kinases						
Sobic.008G015600	AT5G12180	Calcium-dependent protein kinase 17	5.6	6.87E-07		N-terminal CDPK2 signaling triggered enhanced levels of the JA, OPDA and ET, but not SA
Sobic.006G246300	AT4G32300	S-domain-2 5	2.1	5.95E-08	Pathogen^	S-locus receptor protein kinase
Sobic.003G402100	AT5G07280	Leucine-rich repeat transmembrane protein kinase	2.3	5.51E-09		Defense-related processes LRR = roles in stress responses; reception of BR
Hormone- related						
Sobic.003G184900	AT2G23560	Methyl esterase 7	2.3	7.19E-06		
**Plant/Cell Development Regulation**						
Synthases						
Sobic.003G442500	AT4G23990	Cellulose synthase-like G3	2.9	2.33E-07		

Sobic.002G237900	AT1G55850	Cellulose synthase-like E1	2.0	1.18E-07	OPDA+	
Sobic.006G080800	AT2G32540	Cellulose synthase-like B4	5.2	2.77E-08		
Sobic.002G261600	AT1G02850	Beta-glucosidase 11	3.4	0.0001		Degradation of dhurrin
Sobic.009G130000	AT3G23600	Alpha/beta-hydrolases superfamily protein	3.6	5.48E-11		Mediates degradation of cell wall; related to auxin-mediated development of cereal coleoptiles
**Unknown Proteins**						
Sobic.001G371800			2.6	0.0002		
Sobic.002G220000	AT2G31945		2.3	0.0015		
Sobic.002G361001			3.5	1.04E-08		
Sobic.003G234000	AT1G70420		2.4	1.74E-07		
Sobic.003G253700			5.1	5.95E-08		
Sobic.004G114400			2.7	8.53E-07		
Sobic.007G063700			4.2	5.51E-09		
Sobic.007G187800	AT5G61820		3.2	2.61E-06		
Sobic.008G149900			2.1	8.59E-05		

[1] Information about transcripts is based on the exact homologous *Arabidopsis* gene IDs.

[2] Information about transcripts is based on the type of *Arabidopsis* genes.

+ Transcripts reported as upregulated by the treatments or hormones listed.

- Transcripts reported as downregulated by the treatments or hormones listed.

^ Transcripts reported as responsive to the treatments listed, regardless of negative or positive responses.
