# Supplementary material for: Transcriptome Profiling and Genome-Wide Association Studies Reveal GSTs and Other Defense Genes Involved in Multiple Signaling Pathways Induced by Herbicide Safener in Grain Sorghum
Source: Front Plant Sci. 2019 Mar 8;10:192. doi: 10.3389/fpls.2019.00192 (PMC6418823; doi:10.3389/fpls.2019.00192)
Supplement: Supplementary Figure 1 — Safener and herbicide responses in grain sorghum hybrid 7431 seedlings two weeks after treatment. [file Image_1.pdf]

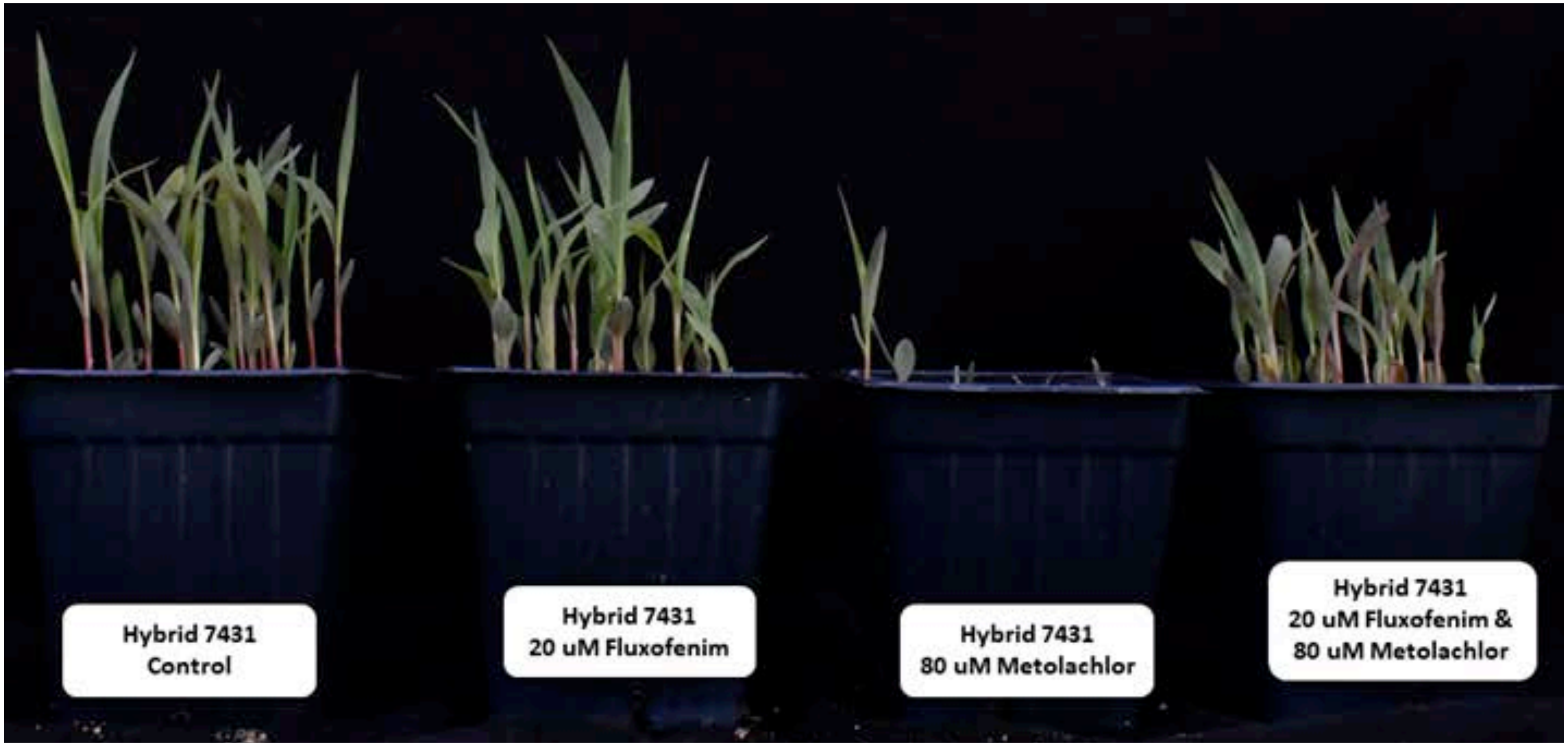

Supplementary Figure 1 | Safener and herbicide responses in grain sorghum hybrid 7431 seedlings two weeks after treatment. From left to right: (1) untreated control (solvents only), (2) 20 µM fluxofenim only, (3) 80 µM *S*-metolachlor only, and (4) 20 µM fluxofenim plus 80 µM *S*-metolachlor. Treatments were applied to each pot via soil drench at 42-hr after planting in 50 mL deionized water. After application, pots were removed from the growth chamber and grown under greenhouse conditions at 28/22ºC day/night with a 16/8-hr photoperiod.

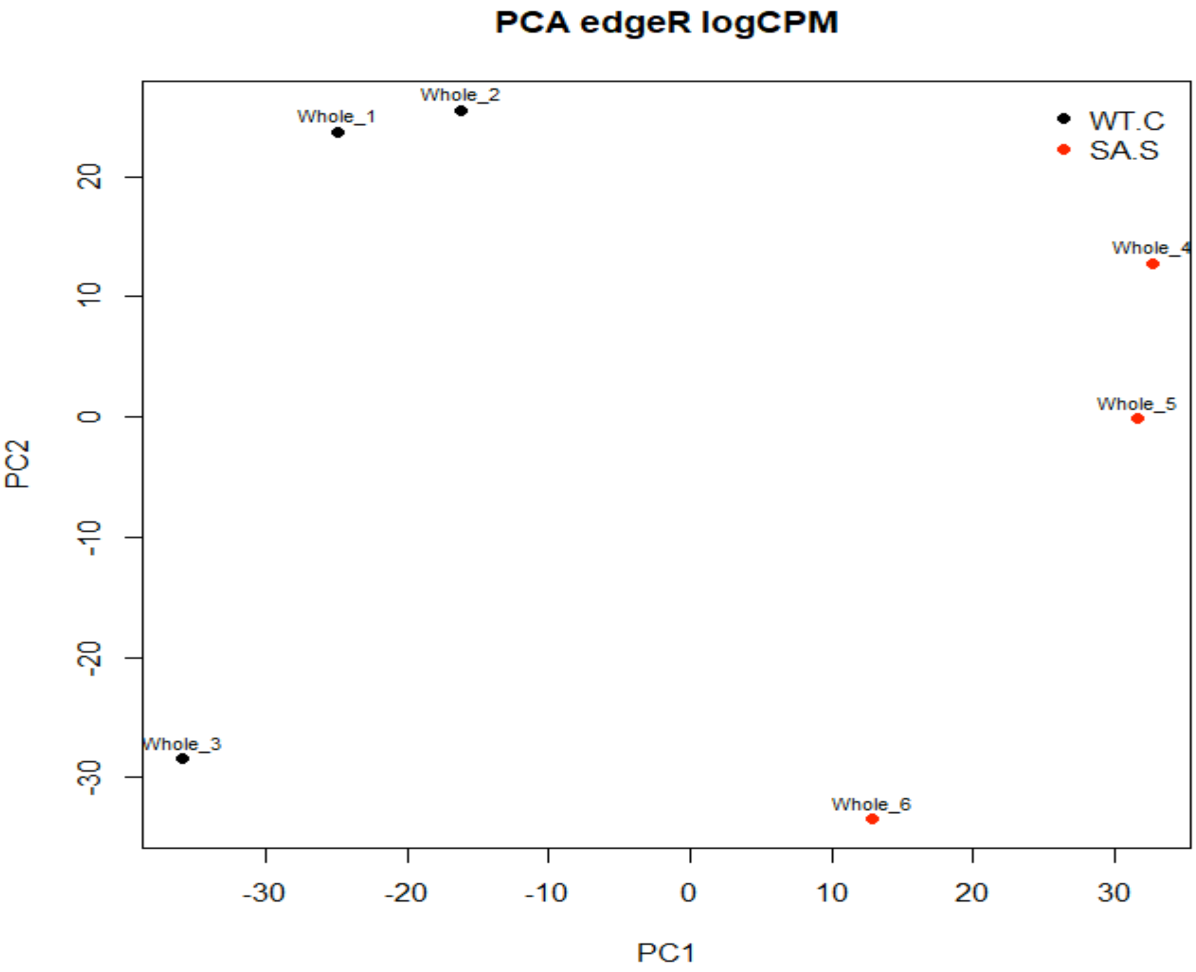

Supplementary Figure 2 | Genome-wide variation in gene expression across six libraries. WT.C (black dots) represents control libraries (0.4% (v/v) dimethyl sulfoxide) and SA.S (red dots) represents safener-treated libraries (20 µM fluxofenim).

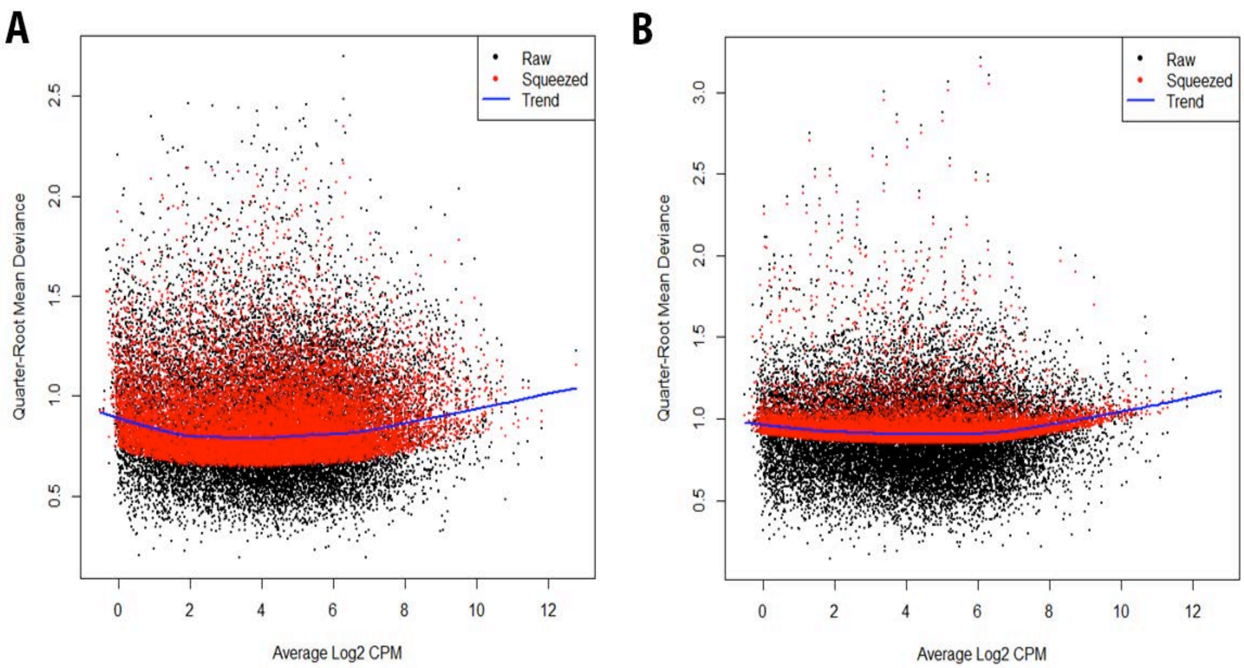

Supplementary Figure 3 | Plots of the quarter-root QL dispersion against the average abundance of each gene before (A) and after (B) adjusting sequencing batches.

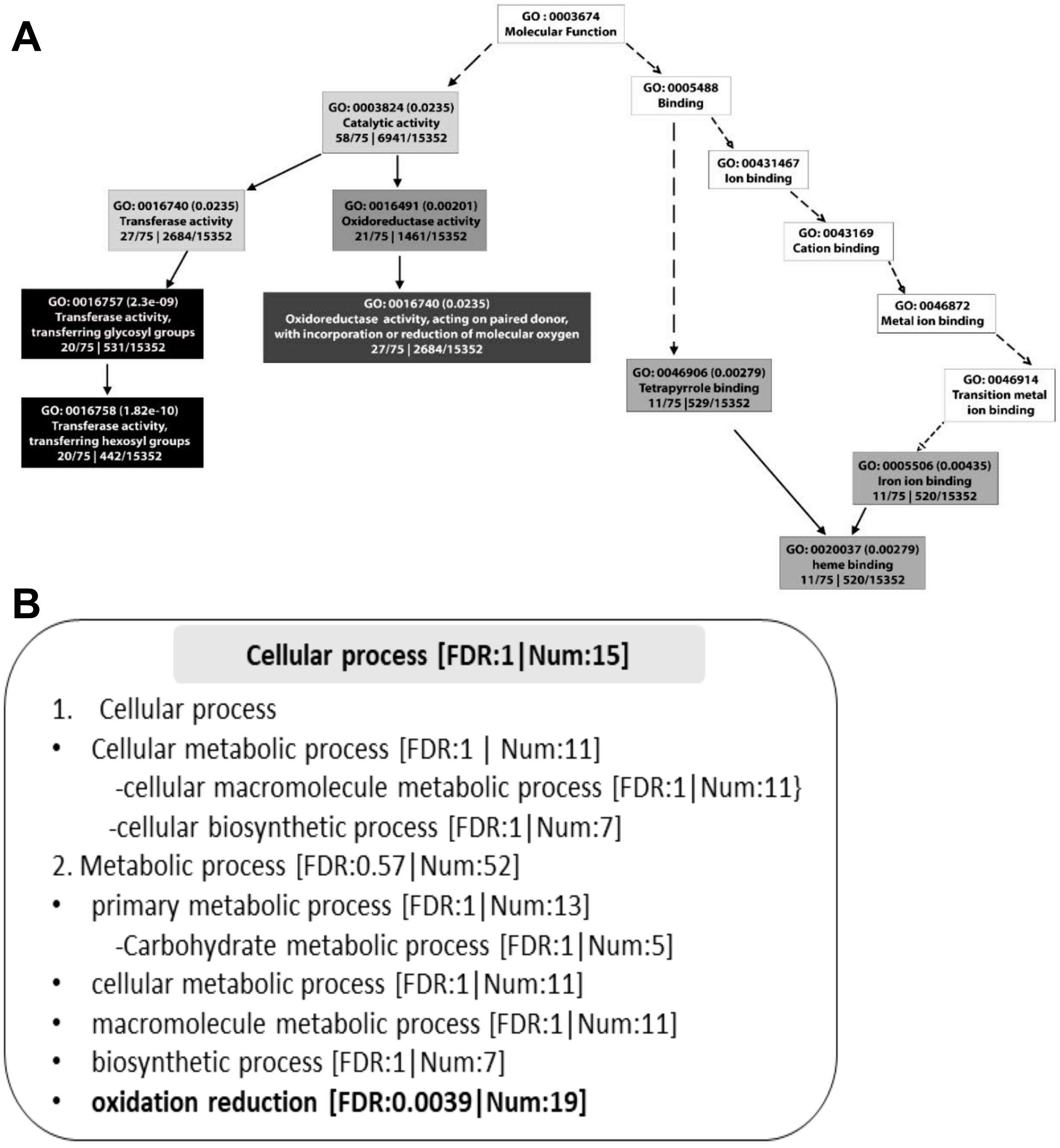

Supplementary Figure 4 | Gene ontology analysis of safener-upregulated genes for molecular (A) and cellular processes (B). The gradation of intensities in each box in (**A**) represents the significance level (darker means more significant); the numbers in the parentheses are the FDR; numbers of genes involved in the function are written at the bottom of each box with the total number of the upregulated genes; in addition the number of genes in the category out of the total number of genes is noted.

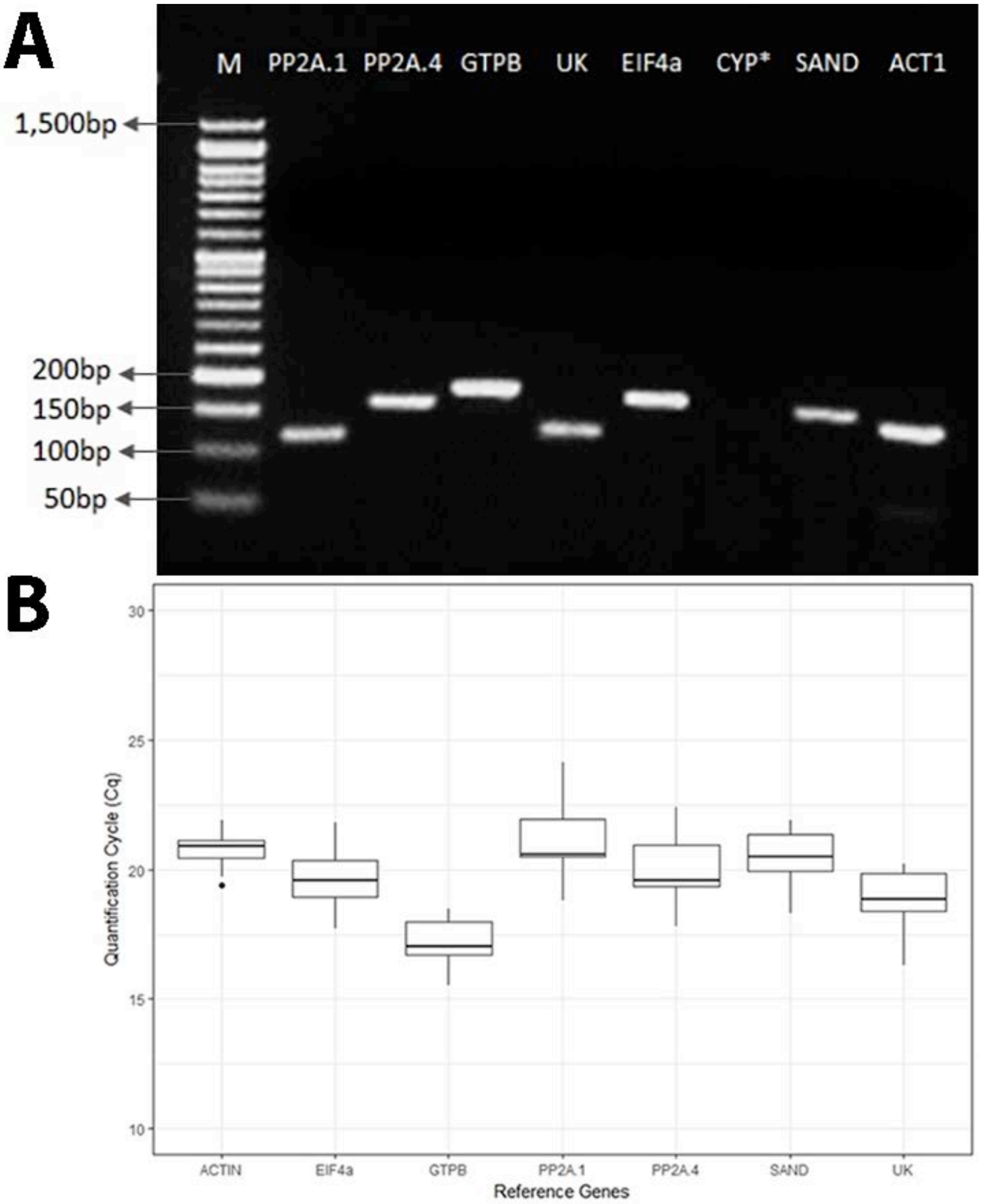

Supplementary Figure 5 | (A) Confirmation of amplicon size and primer specificity for eight candidate reference genes. ****CYP* primers did not produce a PCR product; consequently, this gene was not used in future experiments. M, molecular size markers. **(B) Expression levels of the seven candidate reference genes.** Values are reported as real-time PCR quantification cycle (Cq) values for individual reference genes. Boxes indicate the interquartile rage and the line across the box represents the median. Vertical bars represent the standard deviations of the mean.

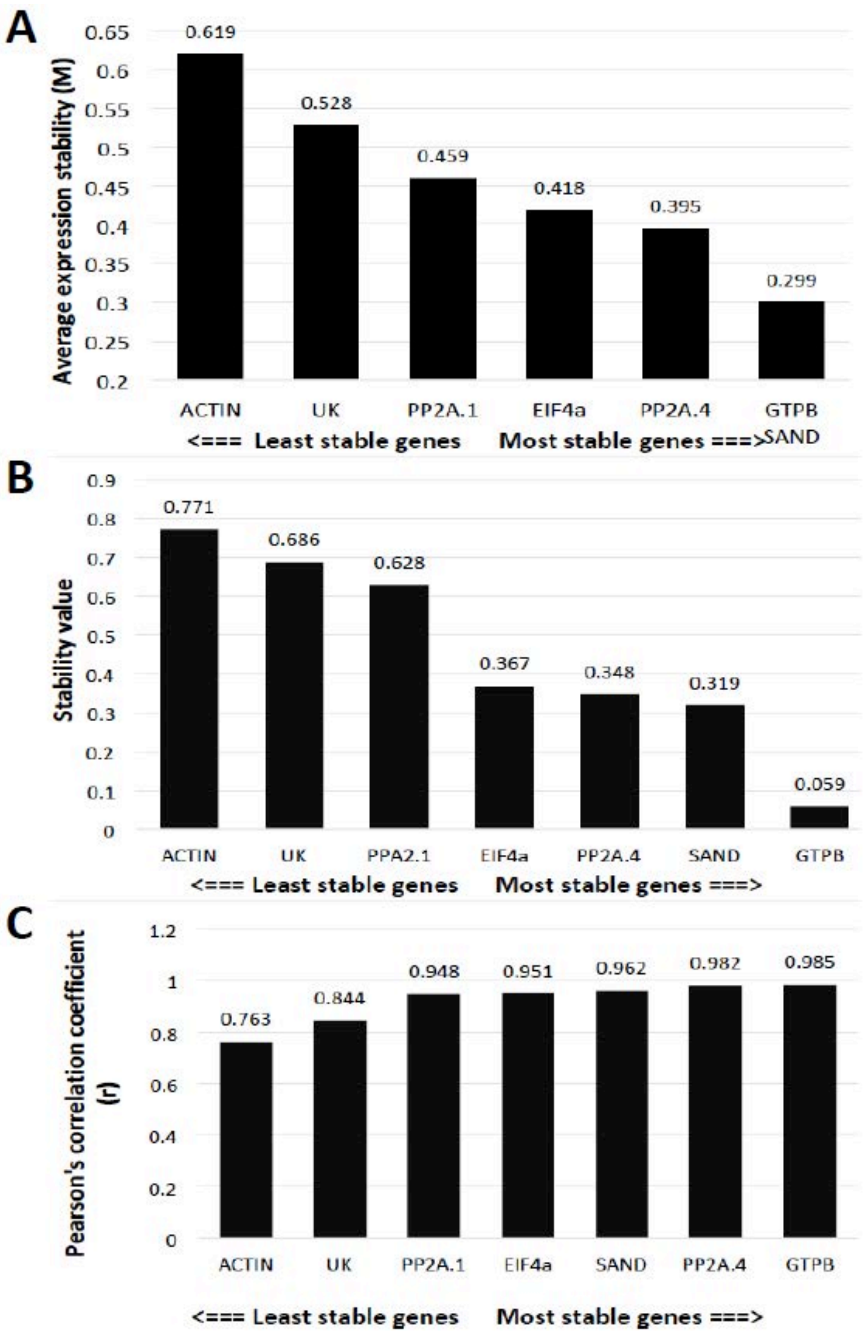

Supplementary Figure 6 | Expression stability and ranking of the seven candidate reference genes calculated by three different evaluation methods. (A) geNorm stability rankings of candidate reference genes based on the average expression stability (M) value for each gene. A lower M value indicates more stable expression. Note that *GTPB* and *SAND* have the lowest, identical M values (0.299). **(B)** NormFinder reference gene stability rankings based on their stability values, which are calculated by combining intra- and inter-group variations for each reference gene. **(C)** Reference gene ranking by gene stability in BestKeeper. Statistical calculations of gene stability are based on correlations between reference genes and the BestKeeper Index. Values are shown as Pearson's correlation coefficients (r).

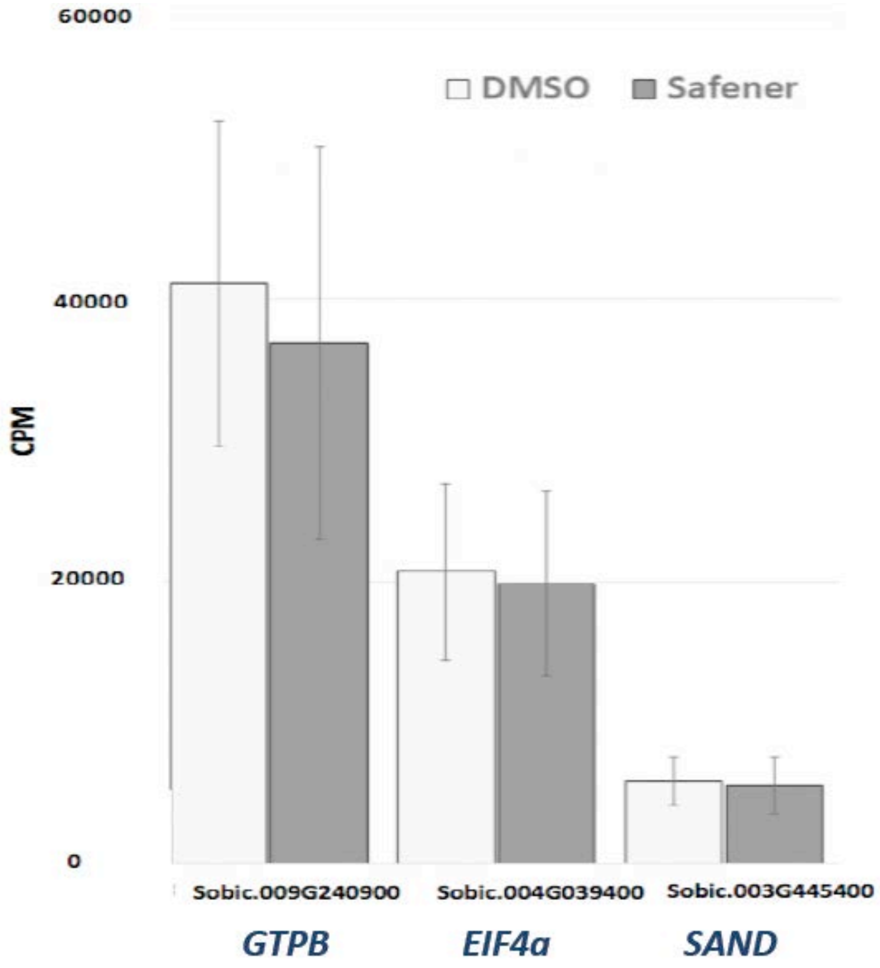

Supplementary Figure 7 | Raw CPM reads of three selected references genes (*GTPB*, *EIF4a*, and *SAND*) by RNAseq analysis at 12-hr after treatment with 20 µM fluxofenim in sorghum hybrid 7431 shoots. Vertical bars represent the standard error of the mean.

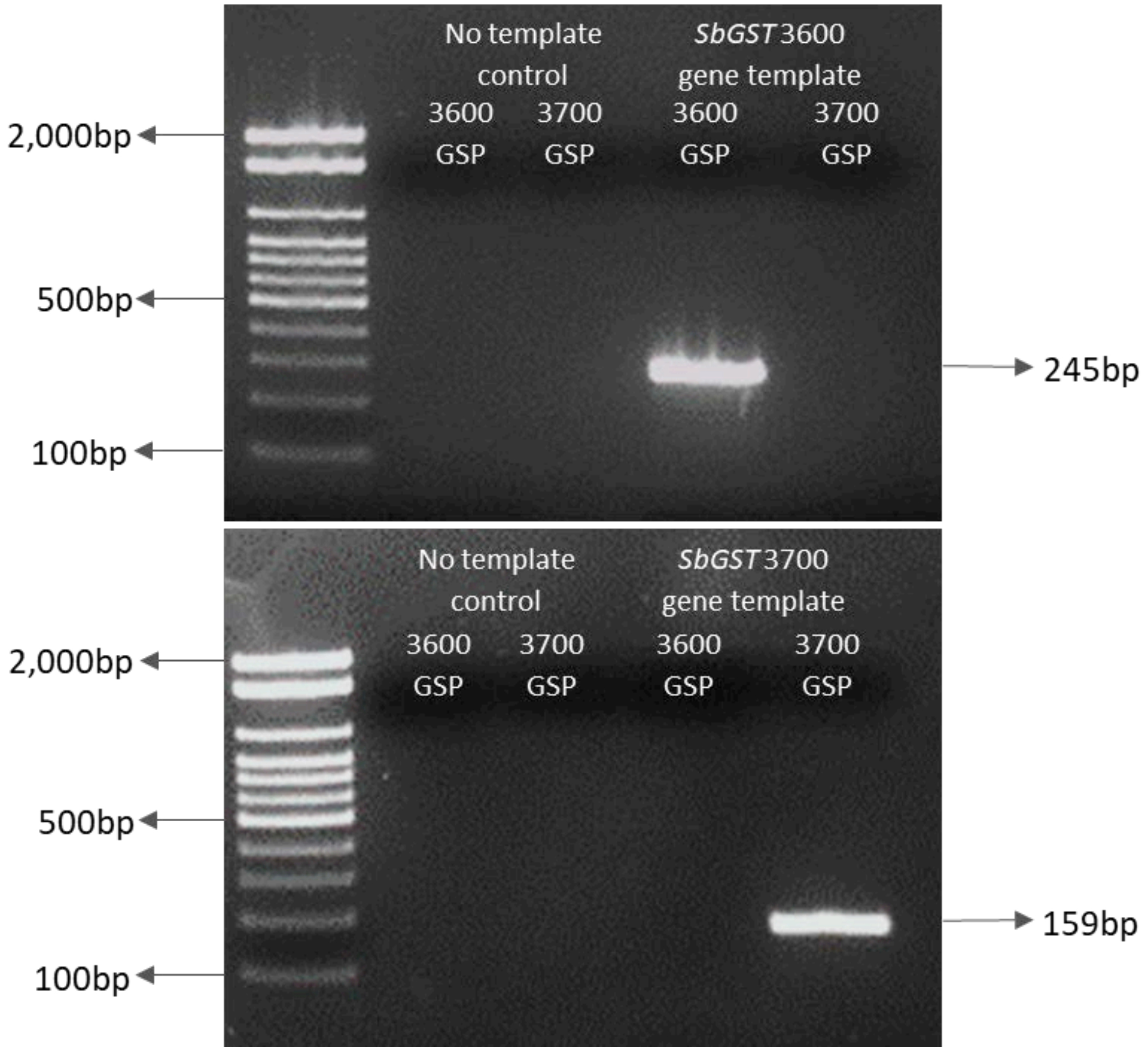

Supplementary Figure 8 | Specificity of glutathione *S*-transferase (*GST*) gene primers. (Top panel) *SbGSTF1* (= *SbGST* 3600) gene template shows a single 245-bp band is present only when amplified by the *SbGSTF1* gene-specific primers (GSPs). **(Bottom panel)** *SbGSTF2* (= *SbGST* 3700) gene template shows a single 159-bp band is present only when amplified by the *SbGSTF2* GSPs. Molecular size markers are shown in the left-hand lane.

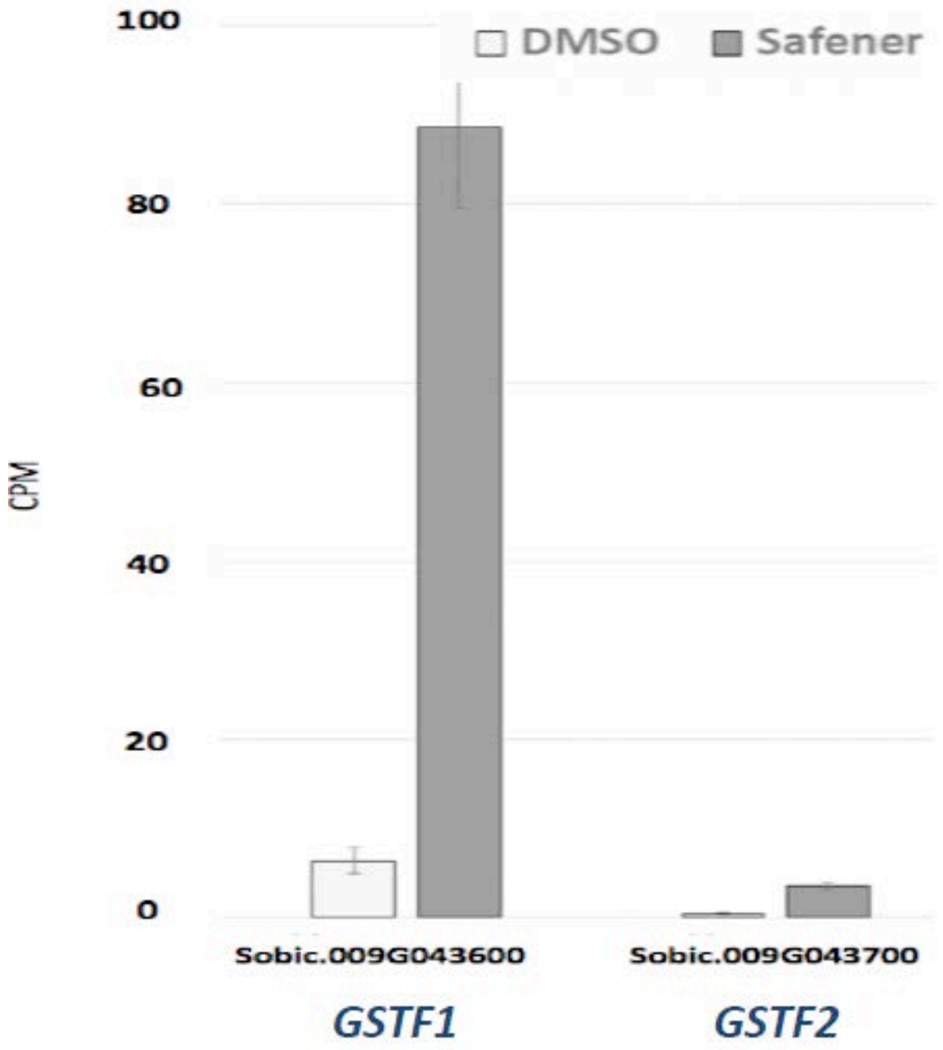

Supplementary Figure 9 | Raw CPM reads from the two tandem *SbGSTs* via RNAseq analysis at 12-hr after treatment with 20 µM fluxofenim in sorghum hybrid 7431 shoots. The actual FC and FDR values compared to the control are listed in **Supplementary Table 2**.

A

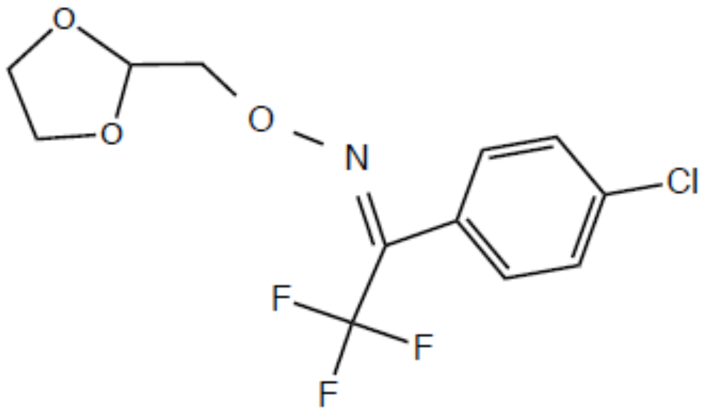

B

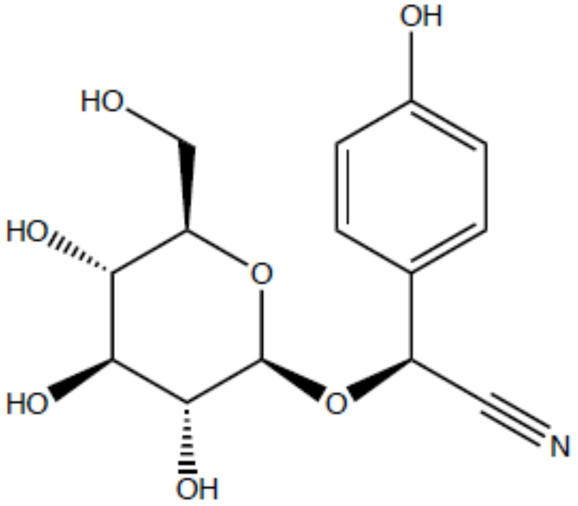

Supplementary Figure 10 | Chemical structures of the sorghum safener fluxofenim (A; an oxime ether**) and dhurrin (B**; a cyanogenic glycoside**).**
